# PIN-LIKES Coordinate Brassinosteroid Signaling with Nuclear Auxin Input in *Arabidopsis thaliana*

**DOI:** 10.1016/j.cub.2020.02.002

**Published:** 2020-05-04

**Authors:** Lin Sun, Elena Feraru, Mugurel I. Feraru, Sascha Waidmann, Wenfei Wang, Gisele Passaia, Zhi-Yong Wang, Krzysztof Wabnik, Jürgen Kleine-Vehn

**Affiliations:** 1 Department of Applied Genetics and Cell Biology, University of Natural Resources and Life Sciences (BOKU), Muthgasse 18, Vienna 1190, Austria; 2 Centro de Biotecnología y Genómica de Plantas (CBGP, UPM-INIA) Universidad Politécnica de Madrid (UPM) - Instituto Nacional de Investigación y Tecnología Agraria y Alimentaria (INIA), Campus de Montegancedo-UPM, 28223 Pozuelo de Alarcón, Madrid, Spain; 3 Department of Plant Biology, Carnegie Institution for Science, Stanford, CA 94305, USA; 4 Fujian Provincial Key Laboratory of Agroecological Processing and Safety Monitoring, College of Life Sciences, Fujian Agriculture and Forestry University (FAFU), Fuzhou 350002, China

**Keywords:** phytohormones, crosstalk, auxin, brassinosteroid, PIN-LIKES, high temperature, root, hypocotyl, growth

## Abstract

Auxin and brassinosteroids (BR) are crucial growth regulators and display overlapping functions during plant development. Here, we reveal an alternative phytohormone crosstalk mechanism, revealing that BR signaling controls PIN-LIKES (PILS)-dependent nuclear abundance of auxin. We performed a forward genetic screen for *imperial pils* (*imp*) mutants that enhance the overexpression phenotypes of PILS5 putative intracellular auxin transport facilitator. Here, we report that the *imp1* mutant is defective in the BR-receptor *BRASSINOSTEROID INSENSITIVE 1 (BRI1)*. Our set of data reveals that BR signaling transcriptionally and post-translationally represses the accumulation of PILS proteins at the endoplasmic reticulum, thereby increasing nuclear abundance and signaling of auxin. We demonstrate that this alternative phytohormonal crosstalk mechanism integrates BR signaling into auxin-dependent organ growth rates and likely has widespread importance for plant development.

## Highlights

• Impaired BR perception enhances PILS5 overexpression phenotypes
• BR signaling increases PILS protein turnover
• BR signaling defines PILS-dependent nuclear abundance and signaling of auxin
• PILS-dependent BR-auxin crosstalk affects organ growth

## Graphical Abstract

**Figure:**
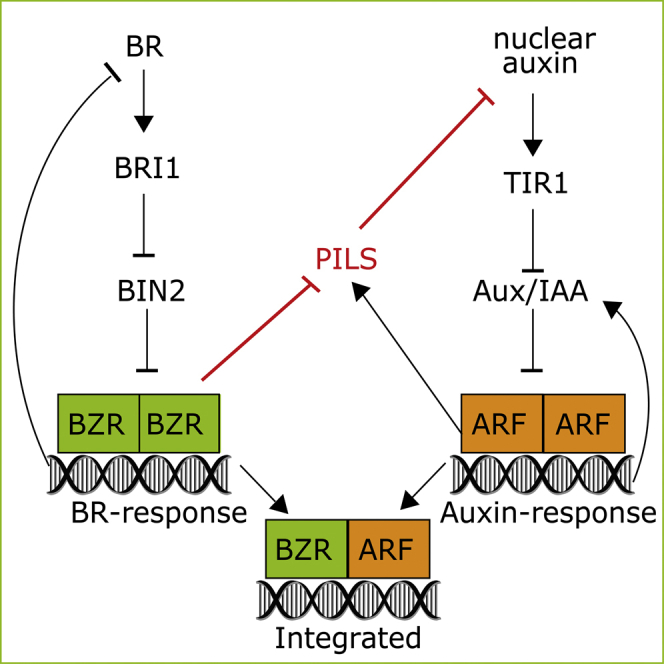

## Teaser

Sun et al. reveal that BR signaling limits the accumulation of PILS proteins at the endoplasmic reticulum, thereby increasing nuclear abundance and signaling of auxin. This alternative phytohormonal crosstalk mechanism integrates BR signaling into auxin-dependent organ growth rates and likely has widespread importance for plant development.

## Introduction

The phytohormone auxin is a key regulator of plant growth and development. Indole-3-acetic acid (IAA), the most abundant endogenous auxin, is perceived by the nuclear F-Box protein TRANSPORT INHIBITOR RESPONSE 1 (TIR1) and its close homologs [1, 2]. Auxin facilitates the binding of TIR1 to its co-receptors of the AUXIN/INDOLE ACETIC ACID (Aux/IAA) family, which initiates the proteasome-dependent degradation of the latter. Subsequently, the AUXIN RESPONSE FACTORS (ARFs) are released from the inhibitory heterodimerization with Aux/IAAs and trigger transcriptional responses [3]. The TIR1 pathway is also involved in rapid, non-genomic responses [4], but the underlying mechanism remains to be elucidated.

Most IAA is synthesized in a two-step biosynthetic route, providing auxin in various tissues [5, 6, 7]. Additionally, plants evolved several mechanisms that are thought to, either transiently (auxin conjugation and conversion) or irreversibly (auxin oxidation and conjugation to certain moieties), modify auxin molecules [8, 9, 10, 11, 12]. These molecular modifications of IAA ultimately abolish its binding to TIR1, thereby directly affecting the nuclear auxin signaling rates [13].

Besides local auxin metabolism, intercellular auxin transport is crucial to define auxin signaling gradients and maxima within plant tissues [14, 15]. The canonical, plasma-membrane-localized PIN-FORMED (PIN) auxin efflux facilitators mainly determine the directionality of intercellular auxin transport and, hence, have outstanding developmental importance [16]. Intriguingly, non-canonical PIN auxin facilitators, such as PIN5 and PIN8, are at least partially retained in the endoplasmic reticulum (ER) and indirectly modulate auxin signaling, presumably through an auxin sequestration mechanism in the ER lumen [17, 18, 19, 20].

In an *in silico* screen, we have previously identified the PIN-LIKES (PILS) protein family of auxin transport facilitators, which resembles the predicted topology of PIN proteins [21]. Despite some structural similarities, the evolution of PIN and PILS proteins is nevertheless distinct within the plant lineage [21, 22]. At the subcellular level, PILS putative auxin carriers control the intracellular auxin accumulation at the ER and restrict nuclear availability and signaling of auxin [21, 22, 23, 24]. Thereby, PILS proteins determine the cellular sensitivity to auxin and contribute to various growth processes during plant development [21, 23, 24].

*PILS* transcription is highly sensitive to environmental conditions, such as light and temperature, integrating external signals to modulate auxin-dependent growth rates [23, 24]. Using a forward genetic screen, we reveal here that *PILS* genes also function as important integrators of endogenous cues, such as brassinosteroid (BR) hormone signaling. Our work illustrates that BR signaling restricts *PILS* transcription and protein levels and, thereby, increases nuclear abundance and signaling of auxin. We conclude that this alternative phytohormonal crosstalk mechanism integrates BR signaling with auxin-dependent organ growth rates.

## Results

### Impaired BR Perception Enhances PILS5 Overexpression Phenotypes

**Figure 1:**
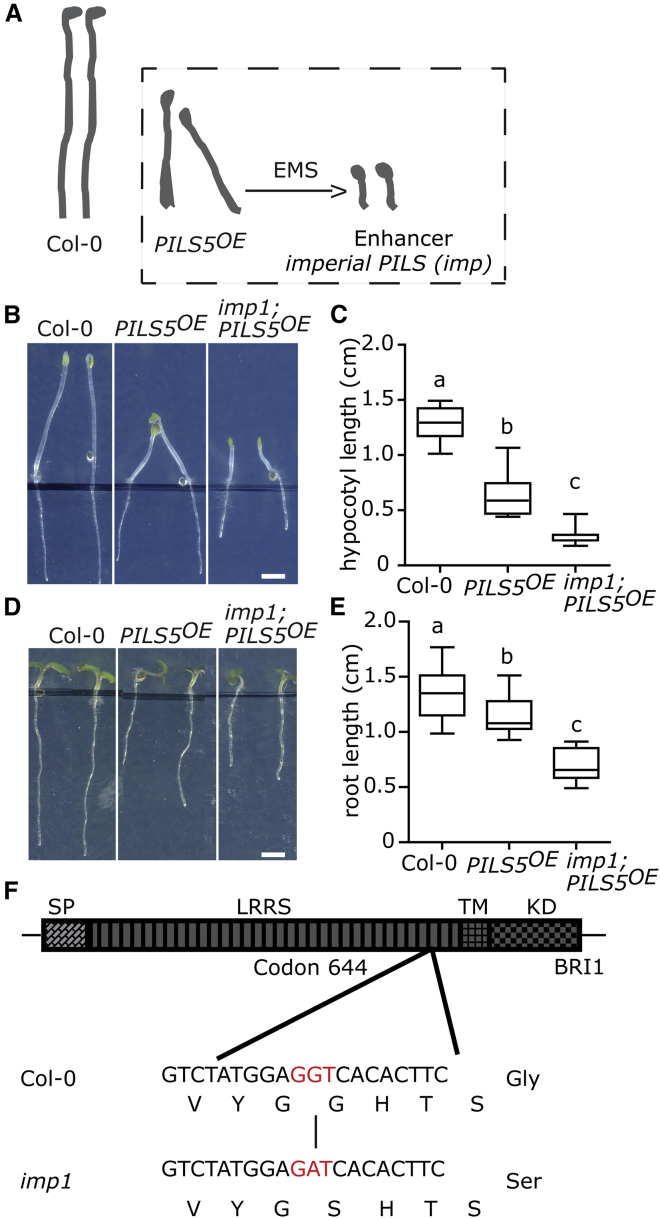
*imp1* Mutation Enhances *PILS5* Overexpression Phenotypes (A) Schematic diagram depicts the “EMS enhancer screen” for identification of genetic modulators of PILS5-related traits. (B–E) Images (B and D) and quantifications (C and E) of 4-day-old dark-grown (B and C) and 6-day-old light-grown (D and E) seedlings of wild-type (Col-0/WT), *PILS5*^*OE*^, and *imp1* mutant grown on ½ MS. Scale bar, 3 mm (B and D). (n > 25). Letters indicate values with statistically significant differences (p < 0.01, one-way ANOVA (C and E)). (F) Sketch of *imp1* mutation in the *BRI1* locus. The diagram shows the full-length BRI1 protein with a defined signal peptide (SP), leucine-rich repeat (LRR), transmembrane (TM), and kinase (KD) domain. The change of G to A in *imp1* results in the conversion of glycine (G) to serine (S) at amino-acid residue 644 in the LRR domain of BRI1.

To assess how intracellular PILS auxin transport facilitators mechanistically contribute to plant development, we performed an unbiased, forward genetic screen. We used an ethyl methanesulfonate (EMS)-mutagenized population of a constitutively expressing PILS5 line (*35S::PILS5-GFP*/*PILS5*^*OE*^) and screened for mutants that either enhance or suppress PILS5-related dark-grown hypocotyl phenotypes (Figure 1A). *PILS5*^*OE*^ seedlings show shorter, partially agravitropic hypocotyls and premature apical hook opening in the dark ([21, 23]; Figures 1B and 1C). From more than 3,000 M1 families, we identified eight *imperial pils* (*imp*) mutants that markedly enhanced the PILS5-related dark-grown hypocotyl phenotypes. Here, we describe the *imp1* mutation, which did not only severely impact on PILS5-dependent hypocotyl growth in the dark (Figures 1B and 1C), but also augmented defects in main root expansion in light-grown seedlings (Figures 1D and 1E), suggesting a broad impact on PILS5-reliant traits.

**Figure 2:**
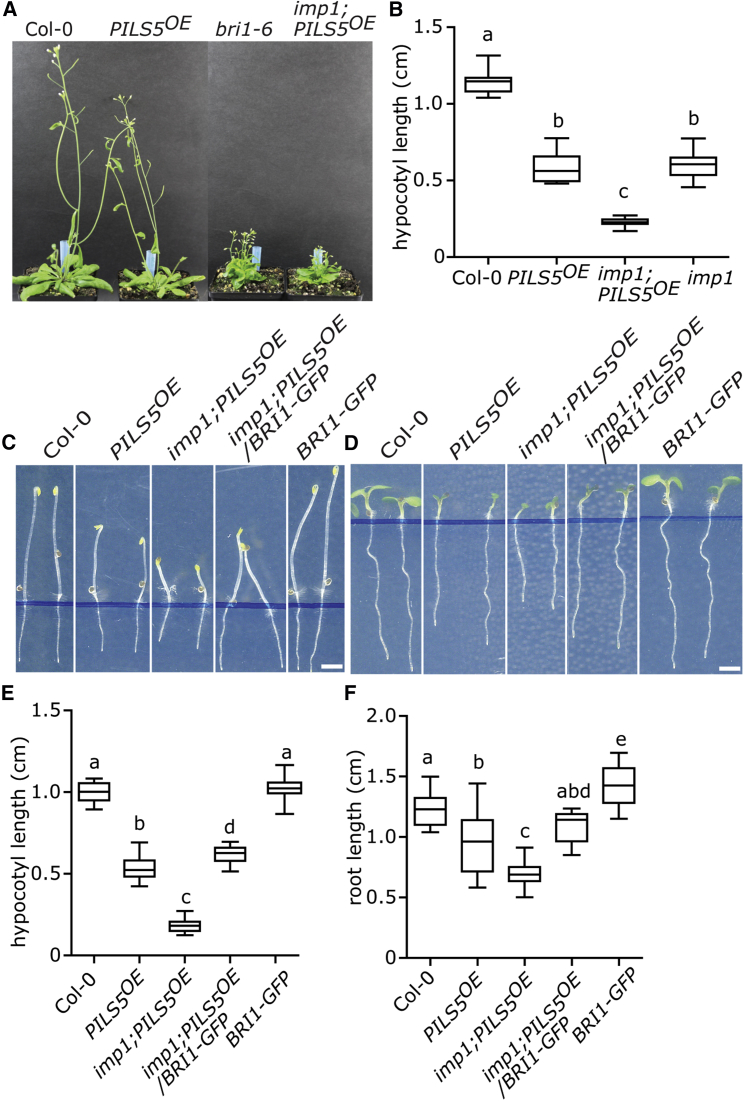
Impaired BR Perception Impacts on PILS5-Related Phenotypes (A) 6-week-old plants of WT, *PILS5*^*OE*^, *bri1-6*, and *imp1;PILS5*^*OE*^ under standard growth conditions. (B) 5-day-old dark-grown hypocotyl quantifications of wild-type, *PILS5*^*OE*^, *imp1*, and *imp1;PILS5*^*OE*^ mutants (n > 25). (C–F) Images and quantifications of 5-day-old dark-grown (C and E, respectively) and 6-day-old light-grown (D and F, respectively) seedlings of wild-type and indicated mutant lines (n > 25). See also Figure S1. Scale bar, 30 mm. W, weeks. Letters indicate values with statistically significant differences (p < 0.01, one-way ANOVA in B, E, and F).

To identify the underlying mutation, we used a combination of classical mapping and next generation sequencing (NGS). During rough mapping, the *imp1* mutation associated within a region of chromosome 4 (18.096 Mb-18.570 Mb), where NGS identified a single mutation (guanine to adenine) that resulted in an amino acid change (glycine [G] 644 to serine [S]) in the BR receptor *BRASSINOSTEROID INSENSITIVE 1* (*BRI1*) (Figure 1F). The identified mutation is reminiscent to the previously isolated partial loss of function alleles *bri1-6* or *bri1-119*, which altered the same site (G644 to aspartic acid [D]) [25, 26]. In agreement, *imp1;PILS5*^*OE*^ rosettes largely resembled the *bri1-6* mutant phenotype (Figure 2A), proposing that the *imp1;PILS5*^*OE*^ mutant impairs BR signaling.

To phenotype the *bri1*^*imp1*^ mutant independently of *PILS5*^*OE*^, we outcrossed the *bri1*^*imp1*^ mutation to *Col-0* wild-type twice and revealed that the *bri1*^*imp1*^ mutant showed a similar reduction in the dark-grown hypocotyl length as *PILS5*^*OE*^, confirming a strong additive effect in *imp1;PILS5*^*OE*^ mutant combination (Figure 2B). Next, we tested the BR sensitivity of *bri1*^*imp1*^ mutant seedlings. Similar to *bri1-6*, the dark-grown hypocotyls, as well as the light-grown roots of *bri1*^*imp1*^ mutant, were strongly resistant to application of 24-Epibrassinolide (BL) (Figure S1A–S1D). These findings confirm that *bri1*^*imp1*^ mutant seedlings are impaired in BR signaling.

To further test whether the absence of *BRI1* enhances PILS5-related phenotypes, we expressed *pBRI1::BRI1-GFP* in the *imp1;PILS5*^*OE*^ mutant background. BRI1-GFP expression indeed complemented the *imp1;PILS5*^*OE*^ mutant, resembling *PILS5*^*OE*^ phenotypes (Figures 2C–2F). These data suggest that the *bri1*^*imp1*^ mutation is responsible for the enhanced *PILS5*^*OE*^-related phenotypes. Additionally, overexpression of *PILS5* in the *bri1-6* and in the *bri1-301* mutant backgrounds largely phenocopied the *imp1;PILS5*^*OE*^ mutant seedlings (Figure S1E–S1J). This set of data suggests that BR perception indeed impacts on PILS5-related traits.

### BR Signaling Modulates *PILS* Gene Expression and PILS Protein Turnover

**Figure 3:**
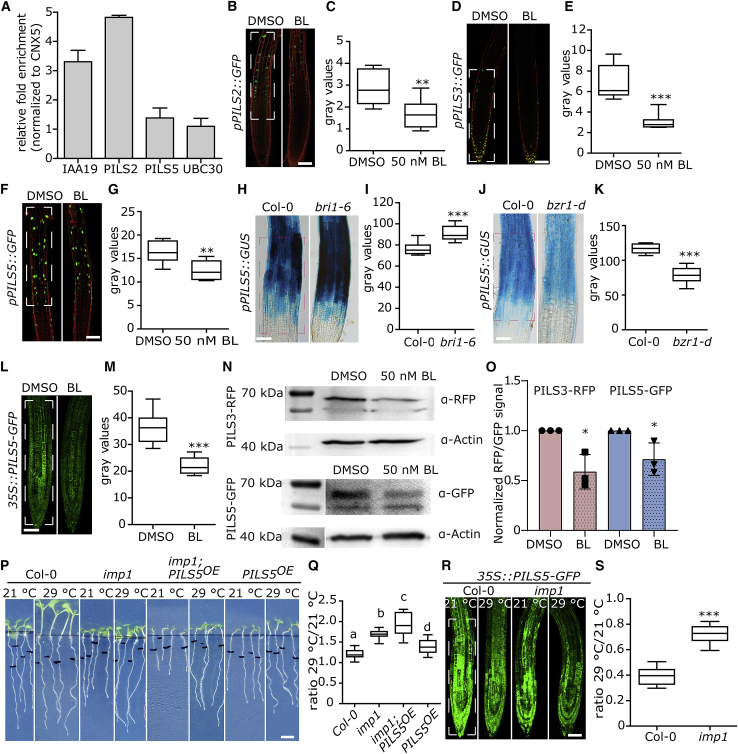
BR Signaling Represses PILS Transcription and Protein Abundance (A) Quantitative assessment of BZR1-CFP binding to promoters of *PILS2* and *PILS5,* using chromatin immunoprecipitation followed by quantitative PCR (ChIP-qPCR). Data represents means ± SD. Representative data of three replicates are shown. See also Figure S2. IAA19 and UBC30 are used as positive and negative controls, respectively. Data are normalized to negative control CNX5. (B–G) Confocal microscopy images (B, D, and F) and quantifications (C, E, and G) of *pPILS2::GFP* (B and C, respectively), *pPILS3::GFP* (D and E, respectively), and *pPILS5::GFP* (F and G, respectively) expression patterns in roots treated with DMSO or 50 nM BL for 12 h (n = 8). Scale bar, 25 μm. (H–K) GUS images (H and J) and measurements (I and K) of *PILS5* promoter activity in main root of *Col-0*, *bri1-6* (H and I, respectively), and *bzr1-d* (J and K, respectively). Scale bars, 25 μm. (L and M) Confocal images (L) and quantification (M) of *p35S::PILS5-GFP* fluorescence after transfer on plates with DMSO or BL for 5 h, showing that BL reduces the PILS5 protein levels in roots of *PILS5*^*OE*^. Scale bar, 25 μm. (N and O) Immunoblot with anti-RFP and anti-GFP antibody (N) and quantification (O) of signal intensity showing that BL downregulates PILS protein levels in *p35S::PILS3-RFP* and *p35S::PILS5-GFP* expressing seedlings. The α-actin antibody was used for normalization. The statistical evaluation shows the differences between the respective DMSO and BL application values. See also Figure S3. (P and Q) Scanned images (P) and quantifications (ratio) (Q) of the root segment grown for 3 days at 21°C and subsequently transferred for another 3 days to 21°C (control) or 29°C (high temperature) (n > 20). Scale bar, 30 mm. (R and S) Confocal images (R) and relative quantifications (S) of *p35S::PILS5-GFP* fluorescence in wild-type and in *bri1* mutant (6 DAG) after exposure to 21°C (control) or 29°C (high temperature) for 3 h (n = 8). See also Figure S3. Scale bar, 25 μm. h, hours; d, days. Stars and letters indicate values with statistically significant differences (^∗^p < 0.05, ^∗∗^p < 0.01, ^∗∗∗^p < 0.001, Student’s t test in C, E, G, I, K, M, O, and S; p < 0.01, one-way ANOVA in Q). The dashed boxes represent the regions of interest (ROIs) used to quantify signal intensity.

We next investigated whether BR signaling modulates *PILS* gene activity, because *in silico* analysis revealed E-boxes (enhancer box) and BRRE-element (BR-response element) [27] in the promoters of *PILS2*, *PILS3*, and *PILS5*, which are potential binding sites for BR-dependent transcription factors BRASSINAZOLE-RESISTANT 1 (BZR1) and BZR2 (Figure S2). Moreover, based on chromatin immunoprecipitation (ChIP)-sequencing, *PILS2* and *PILS5* are direct targets of BZR1 [28, 29]. In agreement, ChIP coupled with qPCR confirmed the BZR1-CFP binding to the promoter of *PILS2* (Figure 3A). In contrast, BZR1-CFP did not associate with the promoter of *PILS5* (Figure 3A), suggesting no or only weak binding. Exogenous application of BL repressed the transcriptional reporters of *PILS2, PILS3,* and *PILS5* fused to green fluorescent protein (GFP) and β-glucuronidase (GUS) (Figures 3B–3G). Furthermore, the endogenous transcript levels of *PILS3* and *PILS5*, but not of *PILS2*, were already detectably reduced after 2 h of BL application (Figure S3A). In agreement, *pPILS5::GFP-GUS* was reduced and enhanced in roots of *BRI1* overexpressing lines and in roots of *bri1* mutant alleles, such as *bri1-5* [26], *bri1-6*, and *bri1*^*imp1*^, respectively (Figures 3H, 3I, and S3B–3E). Moreover, we also detected reduced *pPILS5::GFP-GUS* activity in roots of constitutively active *bzr1d* (Figures 3J and 3K), which suggests that the transcription factor BZR1 negatively impacts on *PILS5* gene expression. Based on these findings, we conclude that BR signaling limits the transcription of *PILS* genes.

In additions, we detected in 3-day-old seedlings higher PILS5-GFP signals under the control of the constitutive 35S promoter in late meristematic regions of *bri1*^*imp1*^ mutants when compared with wild-type roots (Figures S3F and S3G), suggesting an additional, non-transcriptional impact. Notably, the relative difference in PILS5-GFP levels were less apparent in older seedlings (Figure 3R), presumably because of further reduction in the meristem size of older *bri1* mutants. Hence, we next assessed whether BR signaling may also regulate PILS protein turnover, using lines that constitutively overexpress PILS-GFP proteins. Using confocal microscopy, we detected a downregulation of PILS-GFP signals, such as GFP-PILS2, GFP-PILS3, PILS5-GFP, and PILS6-GFP within hours of BL application (Figures 3L, 3M, S3H, and S3I). To confirm that this downregulation is not related to GFP quenching, we also assessed *35S::PILS3-RFP* (fused to red fluorescent protein) and *35S::PILS5-GFP* protein abundance using western blots. In agreement with the confocal imaging, we detected a quantitatively similar BL-induced reduction of PILS3-RFP and PILS5-GFP protein levels (Figures 3N, 3O, S3J, and S3K). This set of data suggests that BR signaling interferes with PILS function in a transcriptional and posttranslational manner.

The dual effect of BR signaling on *PILS* genes and proteins is reminiscent to the impact of high temperature, which also represses PILS proteins in a transcriptional and posttranslational manner [24]. High-temperature-induced downregulation of PILS abundance elevates nuclear auxin input and increases primary root growth [24]. Notably, BRI1-dependent BR signaling is also implied in root growth promotion under elevated ambient temperature [30]. These independent findings prompted us to investigate whether BR signaling and PILS proteins jointly contribute to high-temperature-induced root growth. Both *bri1* mutant and *PILS5* overexpressing line display shorter roots compared with wild-type under standard (21°C) growth conditions, but both lines showed a relative enhancement of root responses to high temperature when compared with wild-type ([24, 30]; Figures 3P and 3Q). The overexpression of PILS5 in *bri1*^*imp1*^ or *bri1-301* mutant background caused a similar, albeit slightly enhanced, root response to high temperature (Figures 3P, 3Q, and S3L–S3N) as compared with *bri1* mutants and *PILS5*^*OE*^*.* The developmental importance of this finding requires further investigations, but our data propose that BRI1 and PILS proteins display overlapping functions in high-temperature-induced root growth. Hence, we next tested whether BR perception modulates PILS abundance under high temperature. We germinated *PILS5*^*OE*^ seedlings at 21°C for 5 days and subsequently shifted the seedlings to 29°C for 3 h. As expected, the PILS5-GFP signal intensity strongly decreased in response to high temperature ([24]; Figures 3R and 3S). In contrast, genetic interference with *BRI1* partially impaired the high-temperature-induced reduction of PILS5-GFP (Figures 3R and 3S). This set of data indicates that BR signaling affects temperature-induced repression of PILS proteins.

### BR Signaling Modulates Auxin Signaling in a PILS-Dependent Manner

**Figure 4:**
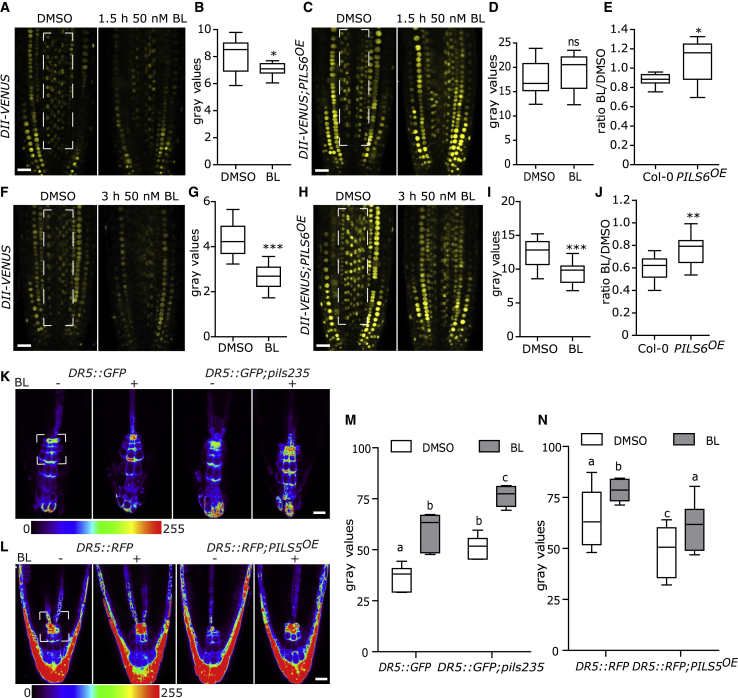
BR Defines PILS-Dependent Nuclear Abundance and Signaling of Auxin (A–J) Confocal images (A, C, F, and H) and absolute (B, D, G, and I) or relative (E and J) quantifications of *DII-VENUS* in wild-type and in *p35S::PILS6-GFP* (*PILS6*^*OE*^) treated with DMSO or 50 nM BL for 1.5 h (A–E) and 3 h (F–J). Scale bars, 25 mm. (K-N) Confocal images (K and L) and quantification (M and N) of *DR5::GFP* in *pils2 pils3 pils5* (*pils235)* (K and M) and *DR5::RFP* in *p35S::PILS5-GFP* (*PILS5*^*OE*^) (L and N) roots exposed to DMSO or 50 nM BL (n > 8). See also Figure S4. Scale bars, 25 μm. Stars and letters indicate values with statistically significant differences (^∗^p < 0.05 ^∗∗^p < 0.01 ^∗∗∗^p < 0.001, Student’s t test in B, D, E, G, I, and J; ns, no significant difference; p < 0.05, two-way ANOVA in M and N). The dashed boxes represent the ROIs used to quantify signal intensity.

BR signaling impacts on ARF transcription factors [31, 32, 33, 34] and modulates auxin signaling output in roots [35]. Notably, the nuclear auxin input marker *DII-VENUS* [36] is also decreased when germinated on BL [37], possibly indicating increased nuclear levels of auxin and, hence, an additional mode of action. In agreement, we noted that even short-term application (within 1.5 h to 3 h) of BL decreased the fluorescence intensity of the *DII-VENUS* (Figures 4A, 4B, 4F, and 4G). This finding indicates that BL exerts a rather direct effect on the nuclear abundance of auxin, proposing an alternative, previously unanticipated BR-auxin crosstalk mechanism.

The ER-localized PILS proteins repress the nuclear availability and signaling of auxin [21, 23, 24, 38], which prompted us to assess next whether BR-induced depletion of PILS proteins defines nuclear abundance of auxin. The BL-induced reduction of PILS6 protein abundance was relatively weak compared with the reduction of PILS3 or PILS5 proteins (Figures 3L, 3M, S3H, and S3I). Hence, we tested whether the constitutive expression of PILS6 could partially counteract the BR-dependent control of nuclear availability of auxin. The BL-induced repression of the nuclear auxin input marker *DII-VENUS* was indeed reduced in *35S::PILS6-GFP* (*PILS6*^*OE*^) line when compared with the wild-type background (Figures 4A–4J). This set of data suggests that the BR-dependent repression of PILS proteins contributes to the modulation of nuclear auxin levels.

The mutated *mDII-VENUS* is the auxin-insensitive version of *DII-VENUS* markers [36, 39], disrupting the interaction between the DII domain, auxin, and the auxin receptors TIR1/AFBs. Prolonged (3 h), but not short-term (1.5 h), exposure to BL treatment induced a partial reduction also in the fluorescence of *mDII-VENUS* (Figure S4A–S4C). This unexpected sensitivity reminds of the high-temperature effect, which also led to strong downregulation of *DII-VENUS* and comparably weaker depletion of *mDII-VENUS* [24]. Previous studies have suggested that mDII is insensitive to auxin [36, 40], but under our conditions, *mDII-VENUS* still remained partially sensitive to BR- (Figure S4A–S4C) or temperature-induced [24] upregulation of nuclear auxin.

Next, we tested if the BR-reliant control of PILS-dependent nuclear abundance modulates auxin output signaling by using the auxin responsive promoter DR5 transcriptionally fused to GFP (*DR5::GFP*; [14]). While the sensitivity of *pils2*, *pils3*, and *pils5* single or double mutant combinations were largely not distinguishable from wild-type, we revealed that the BR-induced auxin signaling was markedly accelerated in *pils2-1 pils3-1 pils5-2* triple mutant roots (Figures 4K, 4M, and S4D–S4F). In conjunction with the BR effect on various PILS proteins, we conclude that PILS proteins redundantly contribute to BR responses. Considering the functional redundancy among the *PILS* genes, *pils2 pils3 pils5* triple mutants are already partially deprived of PILS proteins, which agrees with its hypersensitivity to BR-induced auxin signaling.

As expected, BR-induced repression of PILS5-GFP proteins (Figures 3L and 3M) also correlated with increased nuclear auxin signaling (Figures 4L, 4N, and S4G–S4I). Albeit a similar relative response, the absolute levels of *DR5::RFP* [41] remained quantitatively lower in the *PILS5*^*OE*^ when compared with the respective wild-type seedlings (Figure 4N). This set of data suggests that the BR-dependent repression of PILS5 modulates the nuclear availability and signaling of auxin.

### BR Signaling Modulates PILS-Dependent Organ Growth Rates

**Figure 5:**
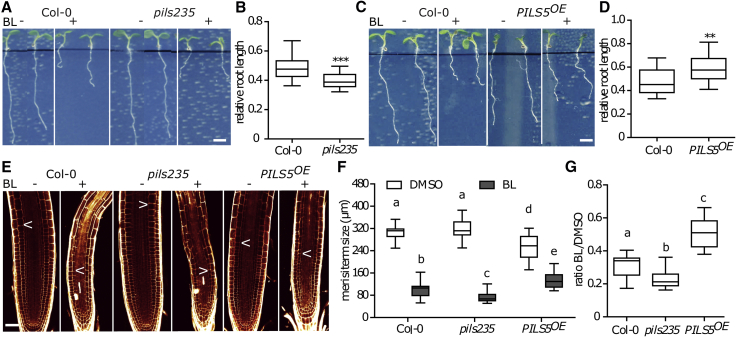
BR Signaling Modulates PILS-Dependent Root Growth (A–D) Images (A and C) and quantifications (B and D) of 6-day-old light-grown seedlings of wild-type, *pils235* (A and B, respectively), and *PILS5*^*OE*^ (C and D, respectively) germinated on plates with DMSO or 50 nM BL (n > 30). Scale bar, 30 mm. (E–G) Confocal images (E) and absolute (F) as well as relative (G) quantification of primary root meristem length of 6-d-old light-grown seedlings germinated on plates with DMSO or 50 nM BL (n = 8). See also Figures S4J–S4L. Scale bars, 25 μm. Stars and letters indicate values with statistically significant differences (^∗∗^p < 0.01 ^∗∗∗^p < 0.001, Student’s t test in B and D; p < 0.05, two-way ANOVA in F; one-way ANOVA in G).

Our set of data proposes that BR signaling represses *PILS* expression and PILS protein abundance, which consequently increases the nuclear availability and signaling of auxin. Thus, we next tested whether PILS proteins could define the root growth sensitivity to BL (Figures 5A and 5B). While the BL-induced root growth repression in *pils2*, *pils3*, *and pils5* single or double mutant combinations were largely indistinguishable from wild-type, we found that *pils2 pils3 pils5* triple mutant roots were hypersensitive to exogenous BL application (Figures 5A and 5B). In contrast, the constitutive expression of PILS5 induced hyposensitive root growth to BL (Figures 5C and 5D). BR perception in the protophloem is sufficient to systemically convey BR action in the root meristem context [37]. On the other hand, BR application limits the cell cycle and, subsequently, root meristem size [42]. In agreement with the root length measurements, the negative impact of BL on meristem size was markedly amplified in *pils2 pils3 pils5* triple mutant and partially restored by constitutive *PILS5* overexpression (Figures 5E–5G). Besides its impact on meristem size, BL application also abolishes radial root patterning [37]. In agreement, BL-induced reduction in root width was enhanced and compromised in *pils2 pils3 pils5* triple mutant and *PILS5* overexpression lines, respectively (Figure S4J). These findings suggest that the BR-dependent control of PILS abundance contributes to root organ growth regulation. Similar to roots, dark-grown hypocotyls of *pils2 pils3 pils5* triple mutant and *PILS5*^*OE*^ showed hyper- and hypo-sensitive growth responses to exogenously applied BL, respectively (Figures S4K and S4L). Accordingly, this set of data proposes that PILS proteins are important integrators of phytohormonal crosstalk, allowing BR signaling to modulate nuclear abundance of auxin. In addition, PILS modulate sensitivity to BL, affecting organ growth.

## Discussion

BRs and auxin play overlapping roles in plant growth and development, and intriguingly, many target genes of BR and auxin signaling are overlapping. Increased auxin levels saturate the BR-stimulated growth response and greatly reduce the BR effects on gene expression [43]. BR-dependent BIN2 signaling component and BZR1/2 transcription factors have been previously shown to directly regulate the ARF transcription factors [31, 32, 33, 34], which are key components in realizing the transcriptional output of auxin. Most intriguingly, BZR1 and ARF6 transcription factors directly interact [33], and this direct crosstalk mechanism is thought to integrate and specify BR and auxin signaling output in shoot elongation. The interaction between auxin and BR in root development is complex, involving positive cross-activation and antagonism that are specific for signaling outputs and cell types [29, 37]. Here, we reveal a higher molecular complexity in the BR-auxin crosstalk, indicating that BR modulates not only auxin output signaling, but also the nuclear input of auxin.

We have previously shown that PILS proteins determine intracellular accumulation of auxin at the ER, decrease cellular sensitivity to auxin, and negatively impact on nuclear availability, as well as the signaling of auxin [21, 23, 24, 38]. Mechanistically, we assume that PILS proteins retain auxin in the ER, and thus reduce the diffusion of auxin from the cytosol into the nucleus. The forward genetic screen presented here reveals that BR signaling restricts the abundance of PILS proteins and, thereby, increases nuclear input and signaling rates of auxin. We, accordingly, revealed an alternative, unanticipated BR-auxin crosstalk mechanism, which may also explain how BR sensitizes seedlings to auxin [31].

Auxin signaling itself stimulates *PILS* gene expression [21], presumably acting as a negative feedback mechanism that control nuclear auxin level. Additionally, external cues, such as light, modulate *PILS* transcription in a PHYTOCHROME INTERACTING FACTORS (PIFs)-dependent manner and, thereby, define differential growth responses in apical hooks [23]. Here, we show that BR signaling directly represses expression of *PILS* genes. Besides the effect on *PILS* transcription, we show that BR signaling posttranslationally restricts PILS protein levels. This finding is reminiscent to an effect of high temperature, which also transcriptionally and posttranslationally limits PILS protein amounts and, thereby, increases nuclear abundance and signaling of auxin [24]. Moreover, high temperature induces root organ growth in a BR- [30] and auxin-dependent manner [24]. Here, we illustrate that high-temperature-induced repression of PILS5 protein requires BR signaling.

**Figure 6:**
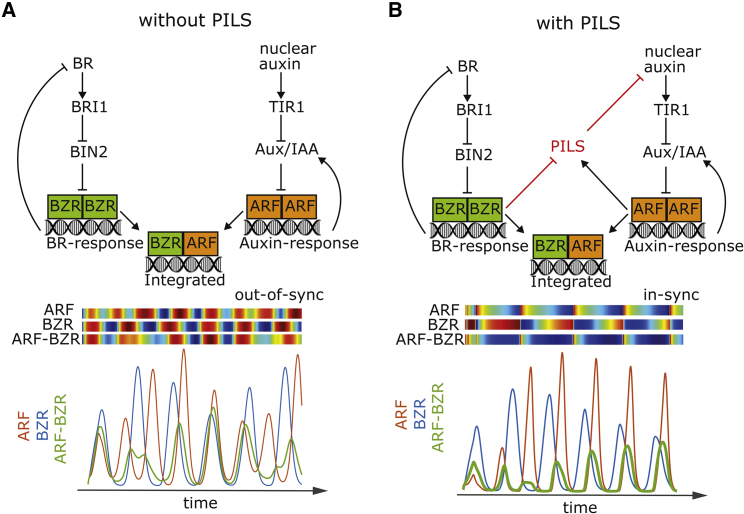
A Computer Model Predicts PILS-Dependent Synchronization of BR and Auxin Responses (A and B) Schematics of BR-auxin oscillatory mechanism without (A) and with PILS-dependent feedback (B) (top panel). Respective computer model simulations are shown as heatmaps (blue to red) and corresponding time-lapse curves with activity peaks for BZR homodimers (blue), ARF homodimers (red), and ARF-BZR heterodimers (green) (bottom panel). See also Figures S5 and S6.

Interestingly, both BR and auxin response pathways are controlled by negative feedback signals [13, 44], which may induce fluctuations in auxin and BR signaling. To further discuss this aspect, we developed a theoretical computer model, simulating the negative feedback on auxin and BR signaling (Figure 6A). In our model, BIN2 limits in a BR sensitive manner the nuclear activity of BZR. Active BZR dimers reduce with delay the BR synthesis, providing the negative feedback on BR signaling. On the other hand, the model incorporates the Aux/IAA repressors of ARF activity. ARF dimers stimulate Aux/IAA levels, initiating the negative feedback on auxin signaling. We integrated the known interaction of ARF and BZR transcription factor to depict the hormonal crosstalk. Due to lack of experimental data, we assumed the same affinity of ARF and BZR for homo- and hetero-dimers. For further details on the mathematical model, please see the detailed description provided in the STAR Methods. The model predicted oscillations of auxin and BR signaling as inferred from ARF and BZR homodimers, respectively (Figure 6A). Dynamic oscillations of auxin signaling contributes to priming of lateral root organs [45], but it remains unknown whether other (or even all) cell types display auxin and/or BR signaling oscillation [46, 47]. In contrast to the homodimers, ARF-BZR heterodimers showed less regular behavior, suggesting that auxin and BR crosstalk signaling are not aligned in this scenario (Figure 6A).

To further discuss our data, we integrated the PILS-dependent BR-auxin crosstalk mechanism into our computer model, assuming a negative transcriptional and posttranslational effect of BR on PILS as well as auxin-dependent regulation of *PILS* genes (Figure 6B; see also detailed model description in STAR Methods). We demonstrate that for estimated parameters, which closely recapitulate experimental data (Figure S5A), the model predicts a strong likelihood of oscillations in ARF/BZR heterodimer formation (Figures 6A, 6B, S5A, S5B, and S6A–6E), suggesting that the here-uncovered PILS-dependent crosstalk mechanism could align auxin and BR signaling outputs. This model output was very robust toward fluctuations in the estimated parameters (Figures S5B and S6A–6E). Notably, the introduction of a positive effect of BIN2 on ARF activity [32] further stabilized the synchrony of auxin and BR signaling in our model (Figures S6D and S6E). On the other hand, reduced (*pils* mutants) and increased (*PILS* overexpression) PILS abundance lessened and enhanced synchrony of ARF/BZR heterodimer signaling, respectively (Figures S6A–6E). While this aspect requires experimental validation, the diverged model behavior is in principle in agreement with distinct responses of *pils2 pils3 pils5* triple mutants and *PILS5*^*OE*^ when challenged with BL (Figures 4K–4N and 5A–5G).

BR does not regulate the expression of PIN intercellular transport components [48], and its effect on root meristem size has been proposed to be independent of auxin [49]. On the other hand, the balance between BR and auxin levels is known to be required for optimal root growth, as these two hormones have different effects on cell division and elongation [29]. The PILS-dependent transverse BR-auxin crosstalk mechanism quantitatively contributes to meristematic activity and overall root growth rates. Untreated *pils2 pils3 pils5* triple mutant and *PILS5*^*OE*^ tendentially display bigger and smaller meristems when compared with wild-type, respectively, consistent with nuclear auxin increasing meristem size. On the other hand, when compared with wild-type, the application of BR reverses the meristem regulation, leading to smaller and bigger root meristems in *pils2 pils3 pils5* triple mutant and *PILS5*^*OE*^ lines, respectively. A similar trend was observed for high temperature-induced root organ growth [24], which likely also involves the here-identified BR-auxin crosstalk. Accordingly, we assume that the BR effect on PILS proteins not only quantitatively set auxin signaling rates, but also qualitatively, define the hormonal crosstalk between BR and auxin. It is also conceivable that the PILS proteins not only mediate BR promotion of nuclear auxin inputs, but also may play a role in auxin-dependent inhibition of BR signaling [29]. Accordingly, we anticipate that BR-dependent control of PILS activity has widespread importance during plant growth and development by synchronizing BR and auxin signaling responses.

## STAR★Methods

### Key Resources Table

**Table:** 

Reagent or Resource	Source	Identifier
**Antibodies**		

Anti-GFP antibody	Abcam	Cat# ab6556; RRID: AB_305564
Anti-RFP antibody	Chromotek	Cat# 6g6-100; RRID: AB_2631395
Actin antibody	Sigma-Aldrich	Cat# A0480; RRID: AB_476670
Anti-YFP antibody	Custom made	N/A
Pierce protein A magnetic beads	Thermo Scientific	88846- #NK80758
Goat anti-mouse	Jackson	Cat# 115-036-003; RRID: AB_2338518
Anti-rabbit	Jackson	Cat# 111-036-003; RRID: AB_2337942

**Chemicals**		

24-Epibrassinolide (BL)	Sigma-Aldrich	E1641

**Experimental Models: Organisms/Strains**		

*bri1-5*	[25]	N/A
*bri1-6* (*Enkheim-2*)	[25]	N/A
*bri1-301*	[50]	N/A
*pBRI1::BRI1-GFP*	[26]	N/A
*bzr1-d*	[44]	N/A
*pDR5rev::GFP*	[14]	N/A
*pDR5rev::mRFP1er*	[41]	N/A
*p35S::PILS2-GFP*, *p35S::GFP-PILS3*	[21]	N/A
*p35S::PILS5-GFP*, *p35S::PILS6-GFP*	[21]	N/A
*p35S::PILS5-GFP;pDR5rev::mRFP1er,*	[21]	N/A
*pils2-1pils5-2*	[21]	N/A
*pPILS2, 3, and 5::GFP/GUS-NLS, pils3-1*	[23]	N/A
*DII-VENUS* and *mDII-VENUS*	[36]	N/A
*DII-VENUS;PILS6*^*OE*^ and *mDII-VENUS;PILS6*^*OE*^	[24]	N/A
*pils2-1 pils3-1 pils5-2*	This study	N/A
*p35S::PILS3-RFP* *pDR5rev::GFP;pils2-1 pils3-1 pils5-2*	This study This study	N/A N/A
*imp1;PILS5*^*OE*^	This study	N/A
*imp1*	This study	N/A
*imp1;PILS5*^*OE*^;*BRI1::BRI1-GFP*	This study	N/A
*pPILS5::GFP/GUS-NLS;pBRI1::BRI1-GFP*	This study	N/A
*pPILS5::GFP/GUS-NLS;bri1-5*	This study	N/A
*pPILS5::GFP/GUS-NLS;bri1-6*	This study	N/A
*pPILS5::GFP/GUS-NLS;imp1*	This study	N/A
*pPILS5::GFP/GUS-NLS;bzr1-d*	This study	N/A
*bri1-6;PILS5*^*OE*^	This study	N/A
*bri1-301;PILS5*^*OE*^	This study	N/A

**Recombinant DNA**		**N/A**

p35S::PILS3-RFP;pK7RWG2	[21]	N/A

**Software and Algorithms**		**N/A**

Graph Pad Prism5	http://www.graphpad.com	N/A
Leica SP5 or Leica SP8	https://www.leica-microsystems.com/	N/A
ImageJ	https://imagej.net	N/A

### Lead Contact and Materials Availability

This study generates new genetic *Arabidopsis* lines (see key resource table). Further information and requests for resources and reagents should be directed to and will be fulfilled by the Lead Contact, Jürgen Kleine-Vehn (juergen.kleine-vehn@boku.ac.at).

### Experimental Model and Subject Details

#### Plant material and growth conditions

*Arabidopsis thaliana* ecotype *Columbia-0* (*Col-0*) and *Landsberg erecta* (*Ler*) were used for experiments. Multiple mutants and marker lines were generated by crossing.

Seeds were stratified at 4°C for 2 days in dark. Seedlings were grown vertically in Petri dishes on ½ Murashige and Skoog (MS) medium supplemented with 1% sucrose and 1% agar (pH 5.9). Plants were grown under the long-day (16 h light/8 h dark) conditions at 21 (±1) °C. For treatments, 5- or 6-d-old seedlings were incubated for 5 h or 12 h on solid and/or in liquid ½ MS medium containing the indicated concentrations of 24-Epibrassinolide (BL) (Sigma; in stock: 1 or 10 mM in DMSO solvent) or germinated for five or six days on MS medium supplemented with BL at 100 nM and 50 nM, respectively. For high temperature (HT)-related experiments, two growth cabinets were equipped with overhead LED cultivation lights (Ikea, 703.231.10), at an irradiance of 150 μmol/m^-2^s^-1^, and set at 21°C (control) or 29°C (HT treatment) under long-day conditions. For microscopy, the seedlings were grown on vertically oriented plates for five days under 21°C, and then kept under 21°C (control) or transferred to 29°C (HT) for 3 h. For root growth analysis, seedlings were grown for seven days under 21°C (control) and for four days under 21°C followed by three days under 29°C (HT).

### Method Details

#### Construction of transgenic plant

Gateway cloning was used to construct *p35S::PILS3-RFP* as described in [21]. The full genomic fragment was cloned into the pDONR221 and 35S promoter region into the pDONR-P4P1, by using the primers listed in Table S1. These entry clones and the RFP-containing entry clone were subsequently transferred to the Gateway-compatible destination vector pK7RWG2 [51]. Transformed lines were selected on kanamycin.

#### Forward genetic screen and mapping

To identify modulators of PILS5, *35S::PILS5-GFP (PILS5*^*OE*^*)* seedlings descended from 3000 ethyl methanesulfonate (EMS) (0.3%) mutagenized M1 plants were analyzed for the dark-grown hypocotyl phenotype. The *imp1* mutant was mapped on the upper arm of chromosome 4 between nga1107 (18.096 Mb) and T5J17-16 (18.570 Mb). A total number of 87 recombinants from the F2 cross between *imp1* (*Columbia* background) and *Landsberg erecta* were used. For *Columbia*/*Landsberg erecta* polymorphism information, the Monsanto *Arabidopsis* Polymorphism and the *Ler* Sequence Collection (Cereon Genomics) were used. For information regarding single nucleotide polymorphisms and insertions/deletions, the *Arabidopsis* Information Resource (TAIR) (http://www.arabidopsis.org) was used.

#### Next generation sequencing

The genomic DNA of *imp1* was prepared for next generation sequencing. Fifteen individuals of F2 progeny derived from cross of *imp1* with the *Col-0* were selected based on the dark-grown hypocotyl phenotype. The selected seedlings were transferred to soil. Subsequently, leaf tissue from 3-w-old plants was harvested for DNA isolation. Genomic DNA extraction was performed using DNeasy plant mini kit (QIAGEN) according to the manufacturer’s handbook. The DNA samples were sent to BGI Tech (https://www.bgi.com) for whole genome Re-sequencing using Illumina’s HiSeq 2000.

#### Phenotype analysis

For hypocotyl analysis, seeds on plates were exposed to light for 8 h at 21°C, cultivated in the dark at 20°C, and scanned at 4- or 5-d-old. For analysis of root length, 6-d-old seedlings on solvent or treatment containing plates were scanned. For root response to HT, 4-d-old root tips of seedlings grown under 21°C were marked before the transfer for three additional days under 21°C (control) or 29°C (HT). Only the root segment grown after the transfer was measured. Plates were scanned with an Epson Perfection V700 scanner. Hypocotyl and root lengths were measured with the ImageJ (http://rsb.info.nih.gov/ij/) software.

#### qRT-PCR analysis

We used roots (cut) from 6-d-old seedlings treated 2 h with or without 50 nM BL and the InnuPREP Plant RNA Kit (Analytic Jena) to extract total RNA. The RNA samples were treated with InnuPREP DNase I (Analytic Jena) before cDNA synthesis. cDNA was synthesized from 1 μg of RNA using the iSCRIPT cDNA Synthesis Kit (Bio-Rad). qRT-PCR was carried out in a C1000 Touch Thermal Cycler equipped with the CFX96 Touch Real-Time PCR Detection System (Bio-Rad), using a Takyon qPCR Kit for SYBER Assay (Eurogentec). All steps were performed according to the manufacturer’s recommendation. We used the *PILS2*, *PILS3*, and *PILS5* gene and *ACTIN2* control primers listed in Table S1. *PILS* genes expression was normalized to the expression of *ACTIN2*.

#### Quantification of root meristem

Root meristems of 6-d-old seedlings grown on solid plates with DMSO or 50 nM BL were imaged with a Leica TCS SP5 confocal microscope. Seedlings were stained with propidium iodide (0.02 mg/mL) (Sigma) before imaging. The meristem size was defined as the distance between the quiescent center and the first rectangular cortical cell [52]. The meristem width was defined as distance between the edges of root meristem. Leica software (LAS AF Lite) was used for quantification.

#### GUS staining

GUS staining was performed and quantified as described previously [53]. The whole seedlings of 5-d-old dark grown or 6-d-old light grown with or without BL treatment were harvested to wells containing 1 mL of cold 90% acetone and incubated for 30 min on ice. The rehydrated seedlings were mounted in chloralhydrate for analysis by light microscopy (Leica DM 5500) equipped with a DFC 300 FX camera (Leica). To quantify the signal intensity, a region of interest (ROI) was defined to capture the most representative signal distribution. This region is indicated in the figures and was kept constant (size and shape) for all analyzed samples.

#### Chromatin immunoprecipitation (ChIP) assays

For ChIP assays, 5 day-old *Arabidopsis* seedlings (pBZR1::BZR1-CFP and negative control 35S::YFP lines) grown in the dark were treated with 100 nM BL (24-Epibrassinolide) for 1 h and cross-linked for 20 min in 1% formaldehyde under vacuum. The chromatin complex was isolated and resuspended in lysis buffer (50 mM HEPES pH 7.5, 1 mM EDTA, 150 mM NaCl, 1% Triton X-100, 0.1% Sodium deoxycholate, 0.1% SDS, 1 mM PMSF, 1X protease inhibitor) followed by sonication to reduce the average DNA fragment size to a range of 200-500 bp. The sonicated chromatin complex was immunoprecipitated using an anti-YFP antibody (custom made) bound to Pierce protein A magnetic beads (Thermo Scientific, Prod #88846, Lot#NK180758). The beads were washed with low-salt buffer (50 mM Tris-HCl pH 8.0, 2 mM EDTA, 150 mM NaCl, 0.5% Triton X-100), high-salt buffer (50 mM Tris-HCl pH 8.0, 2 mM EDTA, 500 mM NaCl, 0.5% Triton X-100), LiCl buffer (10 mM Tris-HCl pH 8.0, 1 mM EDTA, 0.25 M LiCl, 0.5% NP-40, 0.5% deoxycholate) and TE buffer (10 mM Tris-HCl pH 8.0, 1 mM EDTA) and eluted with elution buffer (1%SDS, 0.1 M NaHCO3). After de-crosslinking and DNA recovery, DNA was purified using a PCR purification kit (Thermo Scientific) and analyzed by qPCR. The enrichment of DNA was calculated as the ratio between BZR1-CFP and 35S::YFP samples, normalized to that of the CNX5. Primers for qPCR are listed in Table S1.

#### Western blot

Five-day-old seedlings were transferred for 5 h either on plates with DMSO or 50 nM BL. Root material was ground to fine powder in liquid nitrogen and solubilized with extraction buffer [25 mM Tris, pH 7.5, 10 mM MgCl_2_, 15 mM EGTA, 75 mM NaCl, 1 mM DTT, 0.1% Tween-20, 1% CHAPS with freshly added proteinase inhibitor mixture (Roche)]. After spinning down for 45 min at 4°C with 35,000 x g, the protein concentration was assessed using the Bradford method. Membranes were probed with a 1:5,000 dilution of GFP antibody (ab290, Abcam) or 1:1,000 dilution of RFP antibody (6G6, Chromotek). As loading control, membranes were probe with a 1:2,000 dilution of Actin antibody (A0480, Sigma). Horseradish peroxidase couple goat anti-mouse (115-036-003, Jackson) or anti-rabbit (111-036-003, Jackson) were used as secondary antibodies. The signals were detected and quantified using a Fusion Solo S (Vilber). Samples were used for three independent technical replicates.

#### Confocal microscopy

5- or 6-d-old *35S::GFP-PILS2, 35S::GFP-PILS3, 35S::PILS5-GFP, 35S::PILS6-GFP,* and *pDR5::GFP/RFP* seedlings in *Col-0* or mutant backgrounds were imaged with a Leica SP5 (Leica). Fluorescence signals for GFP (excitation 488 nm, emission peak 509 nm), mRFP1 (excitation 561 nm, emission peak 607 nm) and propidium iodide (PI) staining (excitation 536 nm, emission peak 617 nm) were detected with 20 × (water immersion) or 63 × (water immersion) objective. To image *DII-VENUS* and *mDII-VENUS*, Leica TCS SP8 equipped with a white laser was used, allowing us to separate GFP and YFP fluorophores. The fluorescence signal intensity (mean gray value) of the presented markers was quantified on raw images using the Leica software.

#### Mathematical model description

The dynamics of all components built in the model was simulated using delayed differential equations (DDEs) implemented in MATLAB Inc. The MATLAB-derived dde23 solver (https://www.mathworks.com/help/matlab/ref/dde23.html) was used to obtain direct solutions of DDEs. All simulations were performed until 300 steps to account for multiple oscillations. Overall, our model incorporates BR and auxin signaling pathways [43, 49] with an addition of the here revealed BR-dependent regulation of PILS on transcriptional and post-translational levels.

##### Brassinosteroid signaling branch modeling

BR synthesis is inhibited by its own signaling, which is implemented by BZR dimers (BZR^D^) with delay τ,

(Equation 1) $$\frac{dBR}{dt}=\frac{\alpha_{BR}}{1+k_{BR}\cdot BZR_{\tau }^{D}}-\delta_{BR}\cdot BR$$

where *α*_*BR*_ is BR production rate and *k*_*BR*_ is rate of repression mediated by BZR^D^ and δ_BR_ is a BR turnover rate.

BR perception is known to define BZR activity by inhibiting BIN2 phosphorylation [32]. Hence, we included the BIN2 regulation into our model by,

(Equation 2) $$\frac{dBIN2}{dt}=\alpha_{bin}-BIN2\cdot \left(\delta_{bin}+\delta_{brb}\cdot BR\right),$$

where *α*_*bin*_ and δ_bin_ are BIN2 production and degradation rates and δ_brb_ denotes the rate of BR-dependent BIN2 de-phosphorylation. Next, BIN2 interferes with nuclear BZR activity and thus negatively affects levels of BZR^D^,

(Equation 3) $$\frac{dBZR}{dt}=\alpha_{BZR}-BZR\cdot \left(\delta_{bzr}+\delta_{bzb}\cdot BIN2\right)+{ϒ}_{dB}\cdot BZR^{D}-{ϒ}_{aB}\cdot BZR^{2}+{ϒ}_{dAB}\cdot BZRARF-{ϒ}_{aAB}\cdot BZR\cdot ARF,$$

where *α*_*BZR*_ and δ_bzr_ are BZR production and degradation rates and δ_bzb_ denotes the rate of BIN2-dependent repression of BZR. *γ*_*dB*_ and *γ*_*dAB*_ stand for dissociation rates of BZR^D^ and BZRARF^D^ dimers whereas *γ*_*aB*_ and *γ*_*aAB*_ are association rates of these dimers. Note that BR steers a delayed negative feedback on its own production Equation 1-3.

Furthermore, species of BZR^D^ and BZRARF^D^ are given by following formulas,

(Equation 4) $$\frac{dBZR^{D}}{dt}={ϒ}_{aB}\cdot BZR^{2}-{ϒ}_{dB}\cdot BZR^{D}$$

and

(Equation 5) $$\frac{dBZRARF^{D}}{dt}={ϒ}_{aAB}\cdot BZR\cdot ARF-{ϒ}_{dAB}\cdot BZRARF$$

##### Auxin signaling branch modeling

Nuclear auxin (A) is restricted by PILS auxin transport facilitators [21, 24],

(Equation 6) $$\frac{dA}{dt}=\alpha_{A}-A\cdot \left(\delta_{A}+T\cdot PILS\right),$$

where *α*_*A*_ and δ_A_ are production and degradation constants of auxin and T is PILS transport coefficient.

The dynamics of auxin signaling repressors (AUX/IAA) [46] are modeled by combining ARF- mediated transcription, translation and auxin-dependent degradation in the following formula,

(Equation 7) $$\frac{dAUXIAA}{dt}=\frac{\alpha_{bx}+\alpha \cdot k_{ARF}\cdot ARF_{\tau }^{D}}{1+k_{ARF}\cdot ARF_{\tau }^{D}}-AUXIAA\cdot \left(\delta_{bx}+\delta aux\cdot A\right),$$

where *α*_*bx*_ and δ_bx_ are basal production and degradation constants of AUX/IAA (AUXIAA). α denotes the ARF-dependent transcription rate times amount of ARF homodimers (ARF^D^) and δ_aux_ is an auxin-dependent degradation rate. *k*_*ARF*_ is promoter association constant of ARF^D^. Next, ARF monomers (ARF) are described by the following mathematical equation,

(Equation 8) $$\frac{dARF}{dt}=\alpha_{ARF}\cdot ARF\cdot \left(\delta_{ARF}+\text{θ}\cdot AUXIAA\right)+{ϒ}_{dAF}\cdot ARF^{D}-{ϒ}_{aAF}\cdot ARF^{2}+{ϒ}_{dAB}\cdot BZRAF-{ϒ}_{aAB}\cdot BZR\cdot ARF$$

*α*_*ARF*_ and δ_ARF_ are basal production and degradation rates of ARF monomer and θ represents AUX/IAA-dependent ARF sequestering that leads to negative feedback on AUX/IAA levels. *γ*_*dAF*_ and *γ*_*aAF*_ stand for dissociation and association rates of ARF dimers (ARF^D^) that follow the formula,

(Equation 9) $$\frac{dARF^{D}}{dt}={ϒ}_{aAF}\cdot ARF^{2}-{ϒ}_{dAF}\cdot ARF^{D}$$

Finally, PILS protein levels are coupled to BR and auxin signaling pathway through transcription and degradation and follow this formula,

(Equation 10) $$\frac{dPILS}{dt}=\frac{\alpha_{bp}+\alpha_{PAF}\cdot k_{AF}\cdot ARF_{\tau }^{D}}{1+k_{AF}\cdot ARF_{\tau }^{D}+k_{BZ}\cdot BZR_{\tau }^{D}}-PILS\cdot \left(\delta_{PIL}+\delta_{PBR}\cdot BR\right)$$

where α_bP_ and δ_PIL_ are basal production and degradation rates of PILS proteins, respectively. αpAF denotes the ARF-dependent transcription rate and δPBR is an BR-dependent degradation rate of PILS. kAF is association constant of ARFD to PILS promoter and kBZ is a rate of repression mediated by BZR dimers.

#### Parameter estimation and sensitivity

Parameters of the computer model were estimated by fitting to mean ratios of experimental measurements from triplicates using standard grid search and Monte Carlo sampling to minimize mean squared error between ratios predicted by the model and experimentally observed ratios to fit a linear regression model (Figure S5A). Measurements from four observables that include *PILS* transcription, PILS protein levels as well as auxin response measurements (DR5 and DII reporters, Figure 4) were used.

We used experimental measurements of ratios in PILS5 transcription and PILS5 protein levels from Figures 3D–3M after BL treatments and in BL-related mutants. Similarly, we used measurements of DR5 ratios from three different replicates shown in Figure S4D-4I to fit model parameters (Figure S5A). PILS transcriptional reporter (*pPILS5::GFP*, Figure 3G) was modeled using Equation 10 following the removal of BR-dependent PILS degradation (δ_PBR_ = 0). PILS protein reporter (*35S::PILS5-GFP*, Figure 3M) was modeled using Equation 10 with the experimentally derived estimate of basal transcription rate *α*_*bP*_ = 100 and *α*_*pAF*_ = 0.0, *k*_*AF*_ = 0 and *k*_*BZ*_ = 0. Predicted DR5 transcriptional reporter was modeled as in Equation 7 by removing auxin-dependent degradation δ_aux_ to 0.0 and estimated basal degradation δ_bx_ to 0.075. DII protein levels were modeled following Equation 7 but removing auxin-dependent degradation (α set to 0) and estimating basal production from experiments (3.5 h BL treatments in *pils* and *PILS*^*OE*^; Figures 4G and 4J); *α*_*bx*_ = 75.

BL treatments were modeled by adding constant external source of BR to right hand side of Equation 1; Φ = 0.05 μM. Estimated parameters for *bri1-6* and *bzr1-d* mutants (Figures 3I and 3K) were δ_brb_ = 0.55 and δ_bzb_ = 0.3, respectively. Parameters for *pils* mutant was fitted to measurements of DR5/DII ratios and was *α*_*pAF*_ = 10. BIN2 effect on ARF activation (BIN on ARF) was modeled by reducing AUX/IAA-dependent sequestering of ARFs in Equation 8 such that parameter θ was inversely scaled with BIN2 levels. The reference set of experimentally fitted model parameters is shown below:

**Table:** 

Parameter (Equation)	Estimations Based on Experimental Measurements
*α*_*BR*_ (1)	10 μM/h
*k*_*BR*_ (1)	5 μM
δ_BR_ (1)	0.3 h^-1^
*α*_*bin*_ (2)	10 μM/h
δ_bin_ (2)	0.01 h-1
δ_brb_ (2)	0.7 h-1; 0.55 h-1 (*bri1-6)*
*α*_*BZR*_ (3)	10 μM/h
δ_bzr_ (3)	0.01 h-1
δ_bzb_ (3)	0.5 h-1; 0.3 (*bzr1-d)*
*γ*_*dB*_ (3, 4)	0.5 h-1
*γ*_*dAB*_ (3, 5, 8)	0.5 h-1
*γ*_*aB*_ (3, 4)	1 h-1
*γ*_*aAB*_ (3, 5, 8)	1 h-1
*α*_*A*_ (6)	1 μM/h
δ_A_ (6)	0.01 h-1
T (6)	10 mm/h
*α*_*bx*_ (7)	0.001 μM/h, 75 μM/h (DII-VENUS reporter)
δ_bx_ (7)	0.01 h-1, 0.075 h-1 (DR5 reporter)
δ_aux_ (7)	0.5 h-1, 0 h-1 (DR5 reporter)
*k*_*ARF*_ (7)	0.01 μM
α (7)	1000 μM/h, 0 μM/h (DII-Venus)
*α*_*ARF*_ (8)	10 μM/h
δ_ARF_ (8)	0.01 h-1
θ (8)	0.5 h-1
*γ*_*dAF*_ (8, 9)	0.5 h-1
*γ*_*aAF*_ (8, 9)	1 h-1
*α*_*bP*_ (10)	0.001 μM/h; 100 μM/h (35S::PILS-GFP)
δ_PIL_ (10)	0.01 h-1
*α*_*pAF*_ (10)	1000 μM/h; 0 μM/h (35S::PILS-GFP); 10 μM/h (*pils*)
δ_PBR_ (10)	0.75 h-1; 0 h-1 (pPILS::GFP)
*k*_*AF*_ (10)	0.01 μM; 0 h-1(35S::PILS-GFP)
*k*_*BZ*_ (10)	10 μM; 0 h-1 (35S::PILS-GFP)
τ (1, 7, 10)	10

Finally, key model parameters were varied ± 25% from the estimated values to test model robustness against intrinsic and extrinsic noise (Figure S5B). We could only observe mild alterations of synchrony between auxin and BR signaling that suggests that proposed model is robust.

#### Phase difference calculations - synchrony measure

For each time-dependent solution of ARF^D^ and BZR^D^, amplitudes and periods were calculated, using peak find function (MATLAB Inc.) and subtracted to estimate phase differences between two oscillators. The phase differences were plotted, using violin plot function in MATLAB (https://www.mathworks.com/matlabcentral/fileexchange/45134-violin-plot) together with probability density distributions performed with histfit function (https://www.mathworks.com/help/stats/histfit.html). The large variation in phases (broader distribution) indicates that two oscillatory pathways are out-of-sync, whereas sharper distributions reflect near-perfect synchrony between two signaling pathways.

#### Synchrony of PILS-related mutants and BIN2-mediated ARF activity

One of key findings from our model predictions was that PILS auxin transporters mediate the coupling between BR and auxin signaling pathways, presumably by synchronizing coupled oscillators and maintaining near-constant phase (phase-locking) between oscillations (Figure S6). Next, we consider a model in which ARF activity is promoted by BIN2 (denoted BIN on ARF) as previously suggested in literature [32]. This extended model includes an additional effect of BR signaling on ARF protein activity through BIN2-mediated phosphorylation. Interestingly, we found that such extended model preforms equally or better than the model without this experimentally derived assumption (Figures S6D and S6E).

### Quantification and Statistical Analysis

Hypocotyl and root lengths and GUS intensity (mean gray value) were measured and quantified with the ImageJ (http://rsb.info.nih.gov/ij/) software. The root meristem length, width, and the fluorescence signal intensity (mean gray value) of the presented markers was quantified on raw images using the Leica software (LAS AF Lite). The western blot signals were quantified using a Fusion Solo S (Vilber).

Means and standard errors were calculated and the statistical significance was evaluated using the Graph Pad Prism5 (http://www.graphpad.com) software. The significance of the data was evaluated using the Student’s t test in the case of two columns comparisons. One-way ANOVA followed by Tukey’s test was performed in the case of the multiple columns’ comparisons procedure. Two-way ANOVA followed by Bonferroni post-tests was carried out to compare two different genotypes at different treatments.

Representative data are shown throughout the text. All experiments have been performed in at least three replications.

### Data and Code Availability

This study did not generate/analyze datasets/code.

## Supplemental Information

Document S1. Figures S1–S6 and Table S1

Document S2. Article plus Supplemental Information
