# Supplementary material for: PIN-LIKES Coordinate Brassinosteroid Signaling with Nuclear Auxin Input in Arabidopsis thaliana
Source: Curr Biol. 2020 May 4;30(9):1579–1588.e6. doi: 10.1016/j.cub.2020.02.002 (PMC7198975; doi:10.1016/j.cub.2020.02.002)
Supplement: Document S1. Figures S1–S6 and Table S1 [file mmc1.pdf]

Current Biology, Volume *30*

Supplemental Information

PIN-LIKES Coordinate Brassinosteroid Signaling with Nuclear Auxin Input in *Arabidopsis thaliana*

Lin Sun, Elena Feraru, Mugurel I. Feraru, Sascha Waidmann, Wenfei Wang, Gisele Passaia, Zhi-Yong Wang, Krzysztof Wabnik, and Jürgen Kleine-Vehn

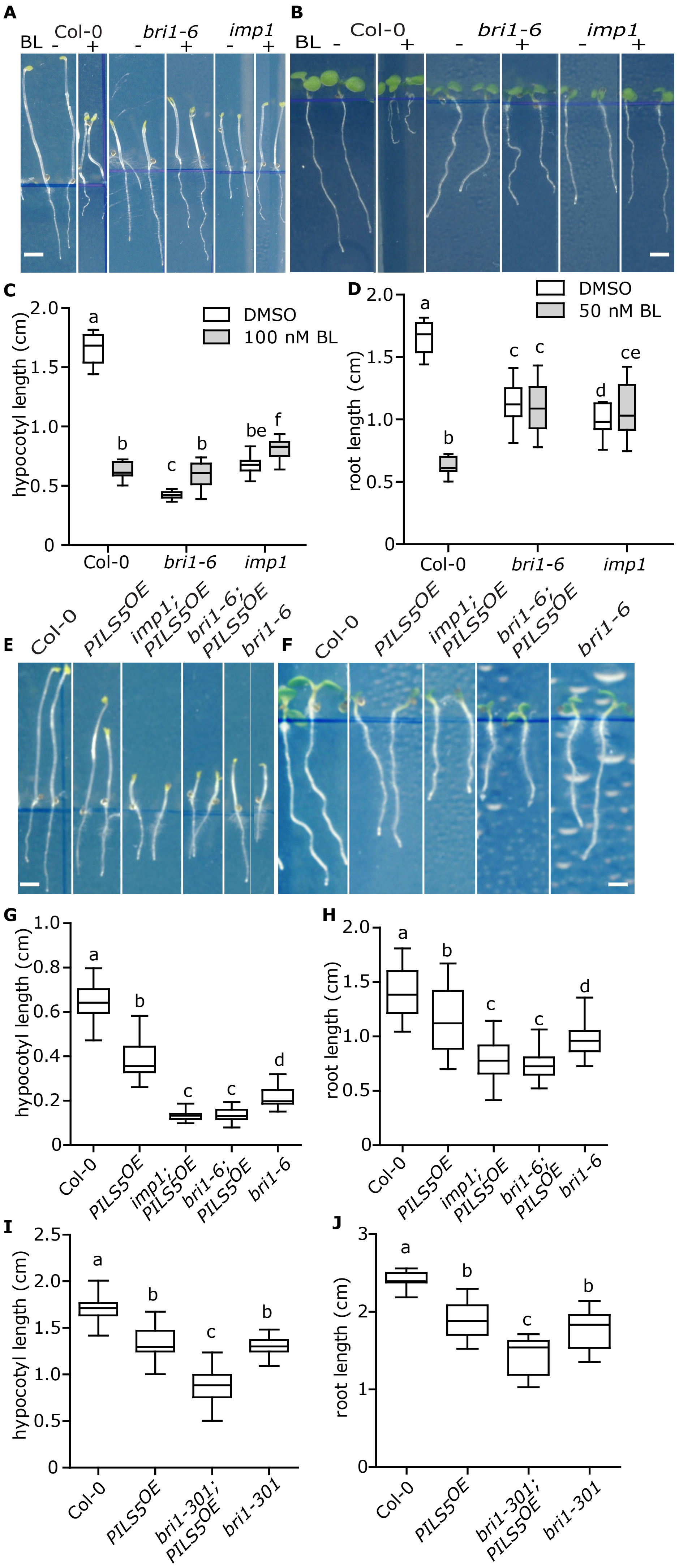

A
BL
Col-0
bri1-6
imp1
- + - + - +
B
BL
Col-0
bri1-6
imp1
- + - + - +
C
hypocotyl length (cm)
DMSO
100 nM BL
a
b
c
b
be
f
Col-0
bri1-6
imp1
D
root length (cm)
DMSO
50 nM BL
a
b
c
c
d
ce
Col-0
bri1-6
imp1
E
Col-0
PILS5OE
imp1; PILS5OE
bri1-6; PILS5OE
bri1-6
F
Col-0
PILS5OE
imp1; PILS5OE
bri1-6; PILS5OE
bri1-6
G
hypocotyl length (cm)
a
b
c
c
d
Col-0
PILS5OE
imp1; PILS5OE
bri1-6; PILS5OE
bri1-6
H
root length (cm)
a
b
c
c
d
Col-0
PILS5OE
imp1; PILS5OE
bri1-6; PILS5OE
bri1-6
I
hypocotyl length (cm)
a
b
c
b
Col-0
PILS5OE
bri1-301; PILS5OE
bri1-301
J
root length (cm)
a
b
c
b
Col-0
PILS5OE
bri1-301; PILS5OE
bri1-301

Figure S1. Impaired BR perception enhances *PILS5^{OE}* phenotypes. Related to Figure 2.

(A-D) Images and quantifications of 5-d-old dark-grown (A, C) and 6-d-old light-grown (B, D) seedlings of wild type (Col-0/WT), *bri1-6* and *imp1* germinated on plates with DMSO, 100 nM (A, C), or 50 nM BL (B, D). (n > 25). Scale bar, 3 mm. (E-H) Scanned images and quantifications of 5-d-old dark-grown (E, G) and 6-d-old light-grown (F, H) seedlings of WT, *PILS5^{OE},* and *bri1* mutants. (n > 25). Scale bar, 3 mm. (I and J) Quantifications of 5-d-old dark-grown (I) and 6-d-old light-grown (J) seedlings of WT, *PILS5^{OE},* and *bri1-301* mutants. Letters indicate values with statistically significant differences (P < 0.05, two-way ANOVA (C and D); P < 0.01, one-way ANOVA (G-J)).

>PILS2 BZR1 BZR2 >89%

ccgccgattcatgcttttaatcttct taaccaattacgtacagacacgagttagtatgtctggttttgggttccacgtggatctgtttggtttgcggcagctattttatt cccacgtgcgattactgatcaaatct
caagccaagcgttccctttttg tcgtcttgttctgatttaatattaaaccggaaatgaccgatcattcagccgctagtgtttggtttgtat atttgacgcattaatgggcttataattggg cttctttttagactc
gtgttgtcgctagcccaatggataatctttgacagtatcgttcaaccacagat tatcttcttcgcacgatgtgataaatacaaa atttcaaatcaagaagaaacttcaagatacaaaattattgcaatcagt
agatttggcgtggatgatgcatctgtcatcgcaatcttgtcttcttcttca ttgacaagttccatgtgtattgtgtagctccaggtgttgttctctaatcggcttta tctctatccaaccatcttcacgactgtggt
ctaattgatttgtt agtgttcttctatttcctggttcagattcttcactgctgtgtatagttcttctcattcaattacttggg ttgtggtttaaatcaccaatttggtcatctt cttgtgtttttttctatcgttgacttcg
agctttacatagtgagatcgag

>PILS3 BZR1 BZR2 >80%

aaagaaattaatgcttgttacttgtattaactcttaa cgaagatcttaatagcaaaacttattaatgatatttcagaaagttggacttttacgacccatttacataacaaagcccattattgtaaactatgggc
tcaatatgtaaggtatattatgaatataatcttaggcccaattaaacacggaagacaacgtaggtttctgcttgtctcgattgttgttgtgtttttatttgttactaaagatagagaaagaaata tcgaagcat
tgtgcgttcatgttcaatatagagttttgttgtaccaaaaaaatatagtgtttgaatctttaattatagactagctagcatataattaacgtgattaactttacaattatggtgatgatgattttctcagtatgt
aggtatagtacttgtcaaagtgacaaataattgcaatttaatgaattggtgtggatccaaaggataacaaatagtatgagcatattcattcgaagcaaaatggtacagaattaagcaaagctttt tgtcca
aaattctaatgaattaaggaccattattttagggaataatagtctttgatgccccacatttccaatagtacaaatcaagattttcaatatatgtcaagtttgttattttcattcacggacattaaaacgagtg
gagcatttcaatttgacatatctttgtccttttctttttgttacagatatcttttgttcacgttctttgaatcatcatattcatagctatgagatcatagaaatatcaaatactgttaattaacc tctttaaaaatac
atattcacaagtctcttgtgtgagtagaaatcctatacaaacaagtttattcatgctctaataagatagataaataagaagatagctcatgacatggccttcattaggcttgttgcatatacaat aagcttac
atataaggaattaaggatggtctaatatctcttgcttcacatgagaagagttataggattcgagtcacataagagatttttattgtagtgtgtataatatatcccacttctacaattatcc agtctattgtgtat
ttttcattaataaatcactaaaatttgcactttgacaatatatacttaaatatttgtatctcgtatatatatatatacatatttttagcactgtatgataattctaattattcgctaattgtttt tttttggtaagaat
tagtcgactattttgatattggggttgttacatttatatcttgcattgaattttatggacggacactggtcgcagcctcacacactaatttgaattttccgtt atcattaatattcgatcgtttcgttggtacgctt
ccatcacacaattaatttaagccaccacactattatcttattctcattcagttttgtttccatttcaaatttcttacgacgcatatgaaaatgtacatttttataacaattccccttaaaaatag atgacaaaaa
gaaaaatgtttttttaaaaaaatatataaaataaaataaataataaatcttcaacaatcgatgaacactttgcaatatctccacac cttgggcaagaaccagaaaaaaacatggggcacgtttcgtagtc
gatgtgacgaaaggcatgacgacggttaccatataaattcgtcttctcagataaagccttattattctcaatagtgttatacgacaatatacgaaacaaacaaagaccccgcttgagttgagttttccaaaa
attgtggaactgtttcctctttctttttgtacataaactaggttacaaaaaattggaaaatatacaaaatcaaaggaca taaacttacgagatatatcataactcataagttgtgaaagttggaattcaaaa
aggagctaactatgaatctatgatacgttattttgttgcttatacggaacaaaacaattccaaaaacaaagaagatgtaaaagtctgacccggatcatgcggacaccaagttgtacatacaaaat atgta
ggatgaaaatatgaaattctcttcccatatgcatagaataataatttaccttagttaaaaagattagtttcta agtttgccccaaataaagagattagtttcttgaaaaaagcattttgtggatagacaaga
atttaaatagaatgaagaggcgaccatgcaagtgttgtactaggccaaattttggagacgctctttctctttcatagagagagagtgagaaagttctgatacggcggcttctcactgtcatctagctgtacc
actgacgataaatctctctcttcctctaaatcg

>PILS5 BZR1BZR2 >90%

ccccagtttgctcttttgtgagccttttctttctttcttggtgtccctcttggtcaccggcgttttcatcccgagatcttgagccatttcgtttggttccagtaatcagcgtagctctgatacca tgttgtgattctca
ctttcactcatgttttgtgaattaaaacgatttagaagttgagagagagatgcataaactctgtttttcttagaattttcccataattctttcttacacacacatacatccttatag caaaacataaagtaaaa
cacaaaatactagcttctgacacctacacgatgacacctacacgattacactgttgcaggctgctcccactacaagcatgcttactctagattatagcttagttttccaagtaaacaaaagcaac tagattc
aaaccaacttctttcacatgtaactacttgttctctctttgacttcttcagtatggtttgaaagcaaaataggcattattcaacagtctgctttctaattaatcctt tgagattgcagtttacgaaaattatccaa
gggattcataattttagttgaagatataaagcaatttccttagcagattacaattacctttagctgttacttgtctgtttagcttcgattggtctagtaaacagttttggtttggattaatcata tttcttattctct
gttaccgttttgttggatctcaaatttctttgatgattctagcaagtggttgagtttaattgatctgtattttatccattaagctgttt tgcagtagcaggtgaaaaacaaacgaattcactgtgttgaatttcatt
caccggaataatcagaggctacgacggcgaaggatgacgatggaaatcttttgattactcatcatacacgtgttgataacatgtggctttgaaaggaattatctgatcaaatcttcttcttcaatattgagtt
tgagttgggatttagtttgttttcgtaactggttatgttgtagggctttttttagcccatgtttgtaatgcttacc tcaataaaatatgaacttgtgagaaaaaaaaaaagatttgggttttagttaaaagagga
gaaagaaatgtttaaaatgatgagctaaattttaaataagttgtaagattaagcaacaaatgagacttgtagaaattgcaaaagttgggacctaaatcttgagaagccactagaataaggacctt atttt
attaatgttgtggcccttgagaaaaataatattgggtaagaaaataaacaacttatagttttcaaaa acacaaaaacacaaaaccctcaatagttggaagagggctccccaagaccgtattcgcgtccag
aatcgtaacattttcgataagattaaaaaaacctgcaaaatctggtccgcacgtttttagcaacacaacaaagtatgacgagaagttccacgatttttgcaggtatagtacattcgcttttcgcc acctcatt
gccttggattcatacactggatcggtttaatcttcttctctgtttttccatgtcaactat tatatagtcattgtatttgtatttattttctatatattgcaccgaaaaaagcaagagagggatatggttcacatga
actatgtaggaggtgaataaattagacacatgtattcttaatttcaggatcacatgaattgtgatagttcggtgctcagtagaacttataaaaaagcacaacacttattctttttgtcggatatg ataatccg
ctttgatttttttaaatatatggagtttcgatagtccaaaaaaa agtttcatgcttaggcagacaaaaactttaatctttgtgtgtggacgacatatttgttttaacttgctagtgaatttttctttaatctac
ctttgttttgtatatatatctaaccattataattatgtatatgttagctgattccaaacgatatcataagttctaatgagtaatgattggtcaatttatcttgttgtaaattgttcaatatacgt gacgtggtccac
ttctatcctccaagactccagctacacaaagacacagtaacacaaacgcatacgcatatgcatgtgtcgtgtctttgtgtgtgtatgttgtatatattaatatattatctaaactataccattttcatgaaattt
ataatatattttctcaaatgatttaagaagataatgatatgtttacgagagtaaaaaaattaatgatcttccagtaatcaataaatatagtaaattgatatcagatcttgttgccaactatagaa ttgaacaa
aatacaaaactgtcatcgccatgttatattatcgtaaagccagcttatatatccttccccattggtgtttttattttttactttgtttggtgaatattaaagcgcttaaaacacgttccaacagaataattgtttg
accttttttagtttatctgattttaatatcaattcttaaaaacatctcctcaatctttgcgtaatttgtcttttcctaatgcatcggatagagatagattcttctgagttcttgcatggttcagg tactttctattcgt
ttcttaatacgtgttctttcttttcggatgaaacataattcatcgatcgatcgatatgtataagtctgataaaaacttgatcgttgcttttgatcattactagtaaaacatctcaataaatcaca taacatgtttc
acgttttgaatcggtttttcagccttgttgtttcagatttgattgatacgaattttaatcattattattgcaaaaattcatatttaaatttgagatgtttttataatatacatgctta agattcataccaataattaa
gttcaataatctagatatggtttagaaactttagatatatagagatgctgatgacatgatgaaggtgatgatcgattgatcgtttattcatttgtcatatatatgagttgtaggaaaaacatctt atgaattta
gatccattaaatacgcttatatgcatgtgtgcatgttcaatgtgtagataagttctattctgaataaattgacgaataacaattattgatttcaatttcgtt ttgagtgtgcatgtcatgtgtctgagatatggg
aaaatgaaaaggaaacttccctatatatataccgaaacaatagactatcaactttgatctatatatacaaattgtagaaaagccttggatctaactgatatttccttatgtgagatacacattaatg tcttttgtt
ttgcttcttaagattaacgtgttatatattatctctttattttaaaacatgttgaccaaaataatttatgtcttacgtgttgcatatga aatagatccaatcgaatcagcctcccatatcttgaatctttgatattt
ggggtctaccattgatatgatttactttgttaattacttgatgttaatatttcatttttcttaagaaattgtaaactaattctttacatatatgcatggcaattaacttagtcaacaggttcaa catttataatgt
atccagaatcccagatagtacctgtcctgataaaaccttttcaggatctcatgatatttcttaaaatata acagcttagtgattttaaataacataaaaattagccgacaaaaagaaaatatcataatataa
ttctgaaaataactaataatttactcttttcaagtaattattcaaaataaatttagaaaactaattcaatgtatttcctcattgtcactcttttatttccctctacaaaattatatatatgaagt agtttttttaaa
atagagccccataactaacattaggggtccatatatttgcattgtaatttata aacacgtgtagaacattataattatttaaaataaaatttagaacaataacttaatgtatttcctcctcgttagtttcctttt
tttccctctacaaaattacatatatggaatagttgttctttacttcttttaagtagagccccttaactaacacattaggggtccatatatttgcattataatttata aacacgtgtagaacctggcactgaagtc
tgatgaatatatatccatttgtttcacaactaccctagct ttttgtatcatcggttactttttttttatcacaagaaatttctcgattttgattttgattttcaatgttgtgaagttgagaagttcaacatgttttg
gacagatgtttttgaacagtaaattagtattttaaacatgtgaatctatttgcaaacgtaatgacgtgtcgtctgaccatttcttgaatgttagttaatttttagctctagttttgattaatct accaattaggtc
attcatttcagtttaagtcaa atcaacaattaggctcgtcctaacgttttcatttagccaaaacaaaaaaaaagatttcaacagtataaataattcgtccttatcaaaaaattaataaataaaaaagatata
catcattacttacgtaattgtttgtattttgatagttttcgataatttctatgtgacggtttttaaaataaaaataaatgttgaataacaaaaaatagtagtacaatagaatactgaattggttt gtgtagggtc
aaaccaacaaatatggaaagaaaagtattatttagttacaacgtgaaaacacaggttcgtgctactactccaatgatacgggtctcaaaatactcaaaactctggcatgtctcaattccacaaattattgt
ttaatcattctgcttcttct

putative BZR1 and BZR2 binding sites predicted by
http://jaspar.genereg.net/

Figure S2. Predicted BZR1 and BZR2 binding motifs in the promoters of *PILS2, PILS3,* and *PILS5*. Related to Figure 3.

Here, we have used JASPAR [54] which is an open-access database of curated, non-redundant transcription factor (TF) binding profiles stored as position frequency matrices (PFMs) and TF flexible models (TFFMs). JASPAR proposed certain probability of BZR binding for *pPILS2* (p > 89%), *pPILS3* (p >80%*),* and *pPILS5* (p > 90%) promoters. The *in silico* predicted G boxes, containing the core E-box motif (ACGT) and/or BZR motif (CGTG), are depicted in yellow (for BZR1) and blue (for BZR2) in *PILS2, PILS3,* and *PILS5* promoter sequences.

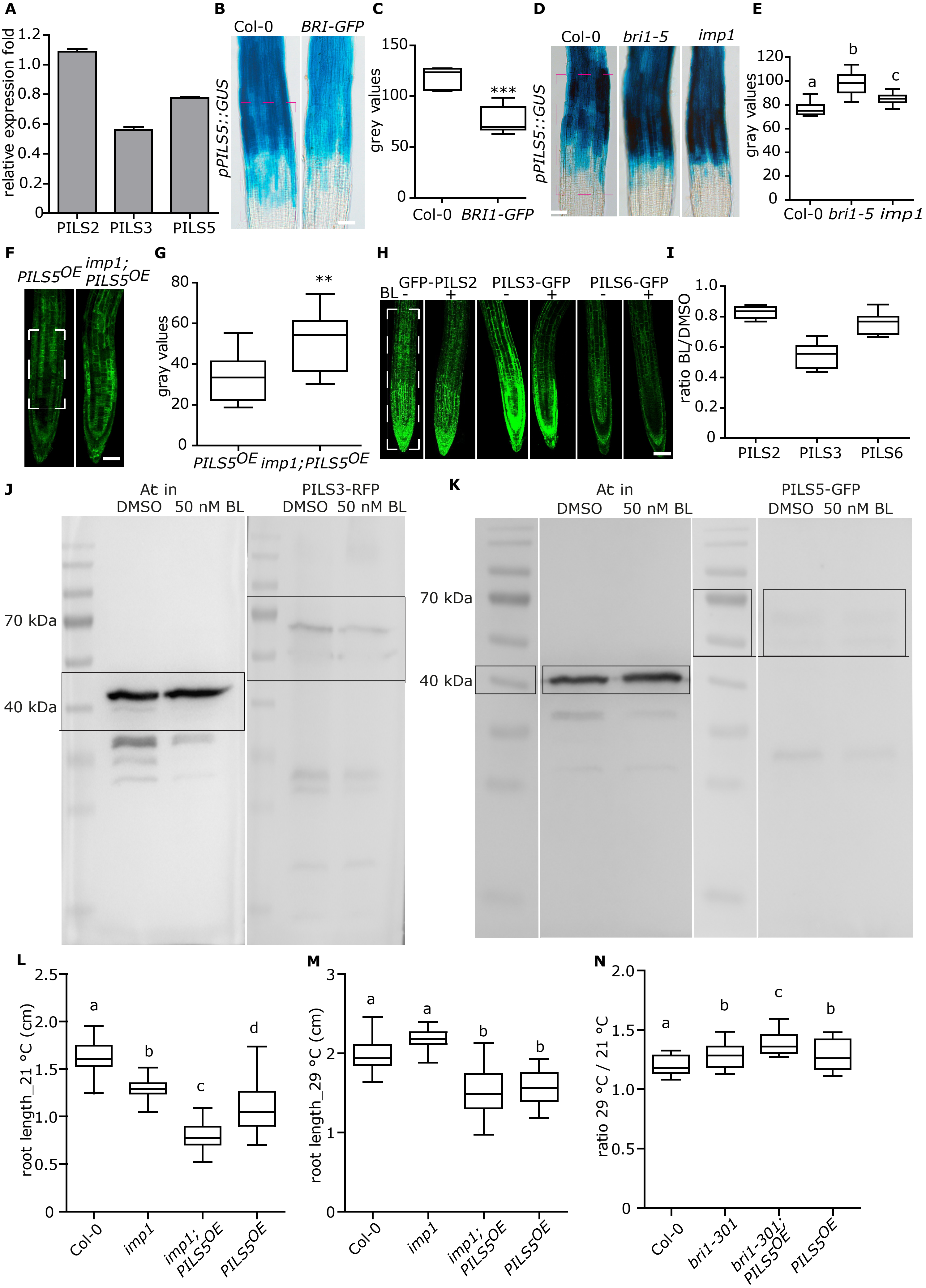
A
relative expression fold
1.2
1.0
0.8
0.6
0.4
0.2
0
PILS2 PILS3 PILS5
B
Col-0
BRI-GFP
pPILS5::GUS
C
150
100
50
0
grey values
***
Col-0 BRI1-GFP
D
Col-0
bri1-5
imp1
pPILS5::GUS
E
120
100
80
60
40
20
0
gray values
a
b
c
Col-0 bri1-5 imp1
F
PILS5OE
imp1; PILS5OE
G
80
60
40
20
0
gray values
**
PILS5OE imp1;PILS5OE
H
GFP-PILS2
PILS3-GFP
PILS6-GFP
BL - + - + - +
I
1.0
0.8
0.6
0.4
0.2
0
ratio BL/DMSO
PILS2 PILS3 PILS6
J
At in
DMSO 50 nM BL
PILS3-RFP
DMSO 50 nM BL
70 kDa
40 kDa
K
At in
DMSO 50 nM BL
PILS5-GFP
DMSO 50 nM BL
70 kDa
40 kDa
L
2.5
2.0
1.5
1.0
0.5
0
root length_21 °C (cm)
a
b
c
d
Col-0
imp1
imp1; PILS5OE
PILS5OE
M
3
2
1
0
root length_29 °C (cm)
a
a
b
b
Col-0
imp1
imp1; PILS5OE
PILS5OE
N
2.0
1.5
1.0
0.5
0
ratio 29 °C / 21 °C
a
b
c
b
Col-0
bri1-301
bri1-301; PILS5OE
PILS5OE

Figure S3. BR signaling negatively impacts on the PILS transcription and protein abundance. Related to Figure 3.

(A) *PILS* gene expression fold changes in 6-d-old light-grown roots of WT after 2 h of 50 nM BL application. (B-E) GUS images (B, D) and quantifications (C, E) of *pPILS5::GUS* expression pattern in roots of wild type, *BRI1-GFP* overexpressor (B, C), *bri1-5*, and *imp1* (D, E). (n = 8). Scale bars, 140 µm. (F and G) Confocal images (F) and quantifications (G) of *35S::PILS5-GFP* in 3-d-old roots of WT and *imp1* mutant. (n = 8). Scale bar, 25 µm. (H and I) Confocal images (H) and quantifications (I) of *35S::GFP-PILS2, 35S::GFP-PILS3*, and *35S::PILS6-GFP,* which were transferred to plates containing DMSO (solvent control) or 50 nM BL for 5 h. (n = 8). Scale bar, 25 µm. (J and K) Unprocessed immunoblot using anti-RFP and anti-GFP antibody. See also Figure 3N, 3O. (L-N) Quantifications and relative quantification of 7-d-old root segments of WT, $PILS5^{OE}$, and *bri1* mutants, which were transferred to 21°C (control) and 29°C (HT) for additional 3 days. (n > 25). The dashed boxes represent the ROIs used to quantify the signal intensity. Stars and letters indicate values with statistically significant differences (**P<0.01, ***P < 0.001, student *t*-test (C and G); P < 0.01, one-way ANOVA (E, L-N)).

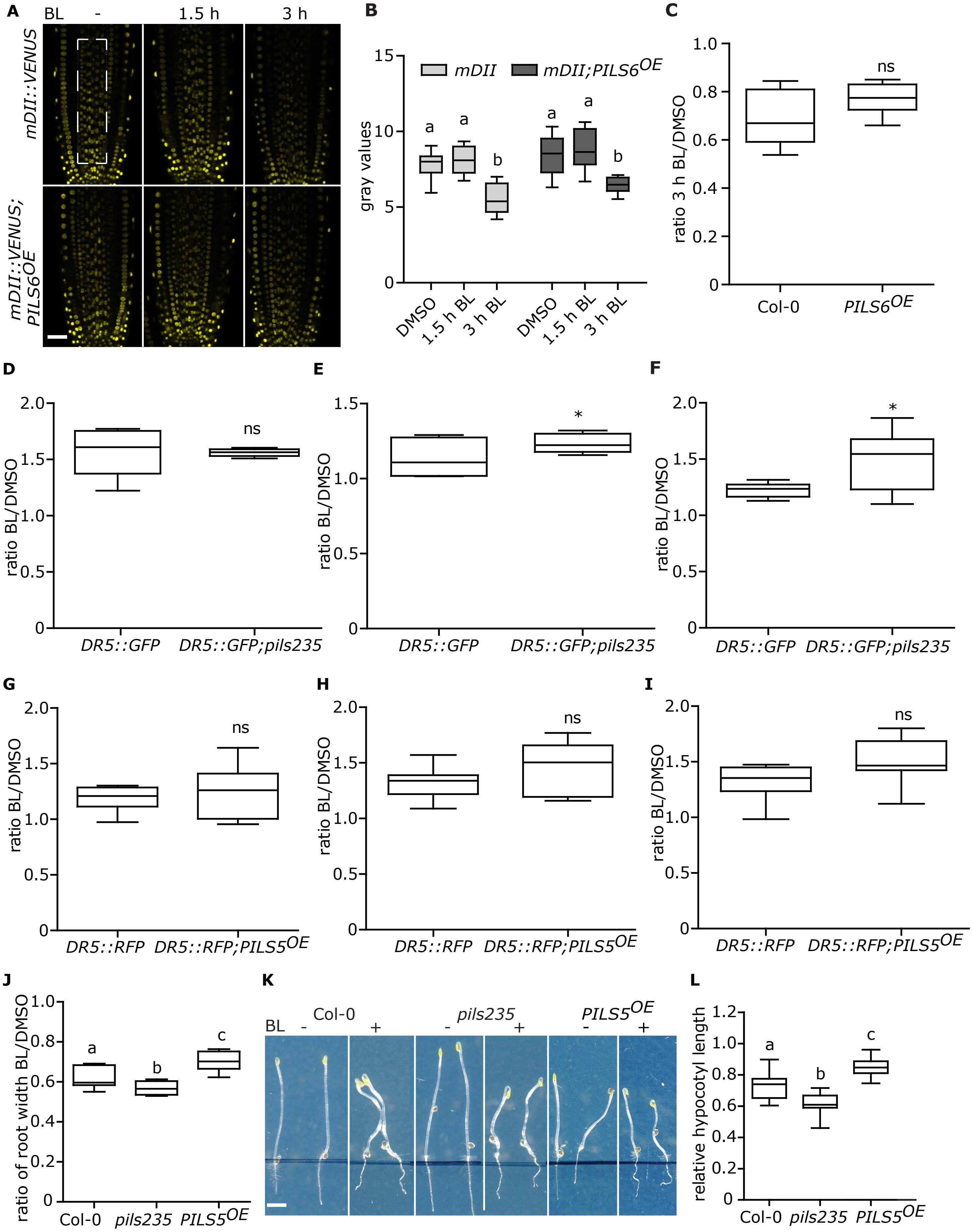

A
BL - 1.5 h 3 h
mDII::VENUS
mDII::VENUS; PILS6OE
B
mDII mDII;PILS6OE
gray values
DMSO 1.5 h BL 3 h BL DMSO 1.5 h BL 3 h BL
C
ratio 3 h BL/DMSO
Col-0 PILS6OE
ns
D
ratio BL/DMSO
DR5::GFP DR5::GFP;pils235
E
DR5::GFP DR5::GFP;pils235
F
DR5::GFP DR5::GFP;pils235
G
DR5::RFP DR5::RFP;PILS5OE
H
DR5::RFP DR5::RFP;PILS5OE
I
DR5::RFP DR5::RFP;PILS5OE
J
ratio of root width BL/DMSO
Col-0 pils235 PILS5OE
K
BL Col-0 pils235 PILS5OE
L
relative hypocotyl length
Col-0 pils235 PILS5OE

Figure S4. PILS proteins define nuclear auxin responses. Related to Figure 4 and Figure 5.

(A-C) Confocal images (A) and quantifications (B, C) of *mDII-VENUS* in WT and in *PILS6*^{OE} after 1.5 h and 3 h of BL application. (n > 8). Scale bars, 25 µm. The dashed boxes represent the ROIs used to quantify signal intensity. (D-I) Quantification (ratios) of *DR5::GFP* (*D-F*) or *DR5::RFP* (*G-I*) in three replicates, showing a relative increase in auxin response in roots of *pils235* (*D-F)*, but no relative difference in *PILS5*^{OE} (*G-I*) when treated with 50 nM BL. (n = 8). D and G is the ratios of Figure 4M and 4N, respectively. (J) Quantification of root widths in the meristematic region of 6-d-old light-grown WT, *pils235*, and *PILS5*^{OE} roots. (K and L) Scanned images (K) and quantifications (L) of 5-d-old dark-grown hypocotyls of WT, *pils235,* and *PILS5*^{OE} germinated on plates with DMSO or 100 nM BL. (n > 25). Scale bar, 3 mm.

Letters and stars indicate values with statistically significant differences (*P<0.01, student's *t*-test (C-I), ns: no significant difference; P < 0.01, one-way ANOVA (B, J, and K)).

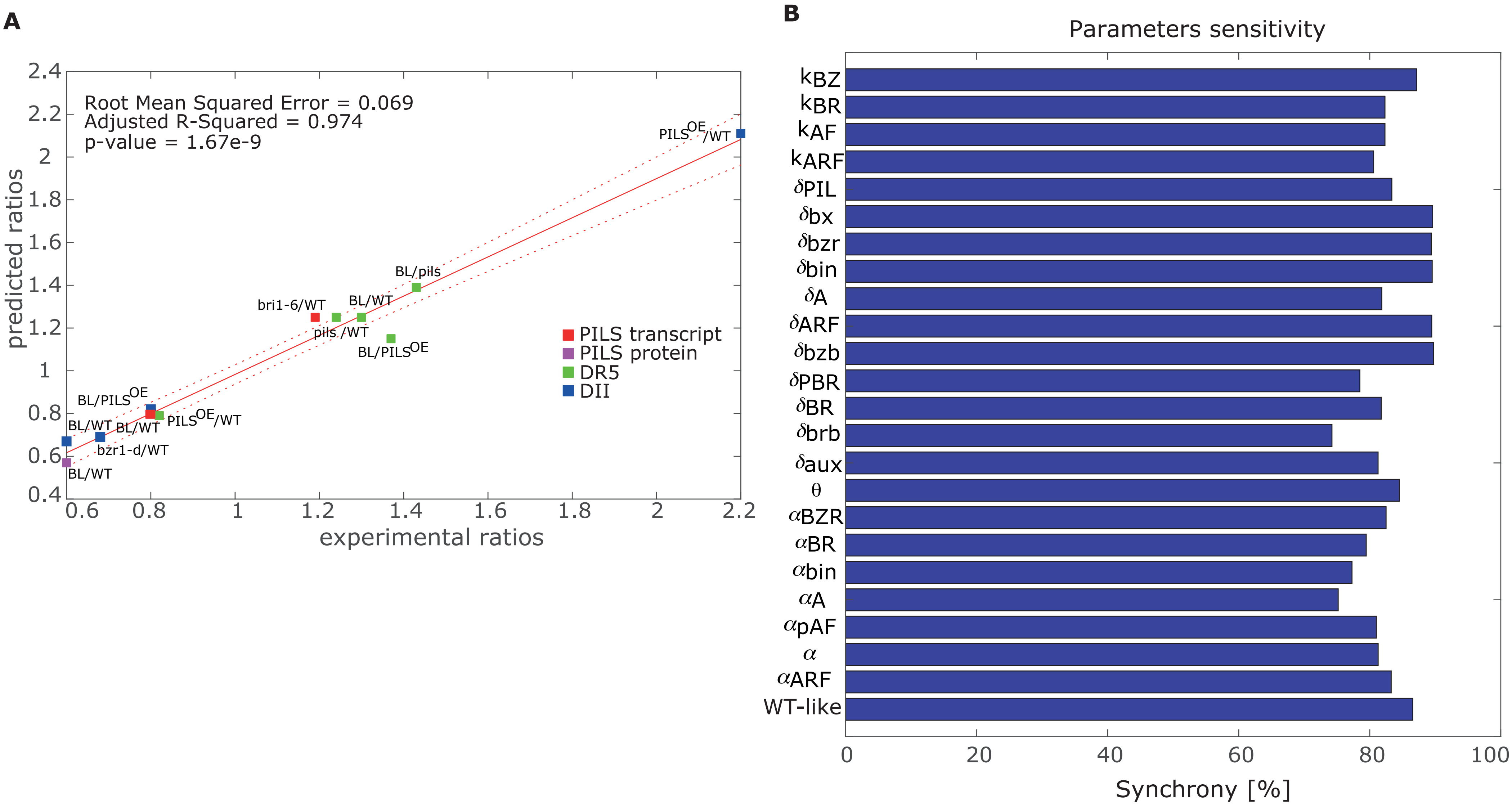
A
Root Mean Squared Error = 0.069
Adjusted R-Squared = 0.974
p-value = 1.67e-9
predicted ratios
experimental ratios
PILS transcript
PILS protein
DR5
DII
PILSOE/WT
BL/pils
bri1-6/WT
BL/WT
pils/WT
BL/PILSOE
BL/PILSOE
BL/WT
BL/WT
PILSOE/WT
bzr1-d/WT
BL/WT
B
Parameters sensitivity
kBZ
kBR
kAF
kARF
δPIL
δbx
δbzr
δbin
δA
δARF
δbzb
δPBR
δBR
δbrb
δaux
θ
αBZR
αBR
αbin
αA
αpAF
α
αARF
WT-like
Synchrony [%]

Figure S5. Estimation of model parameters and testing of model robustness to noise. Related to Figure 6.

(A and B) Depict parameter estimation fitness plot. Each predicted ratio was compared to experimental observations to fit a global linear regression model (A) and model robustness based on +/- 25% variation of parameters (B). Synchrony of auxin and BR signaling was plotted against each parameter.

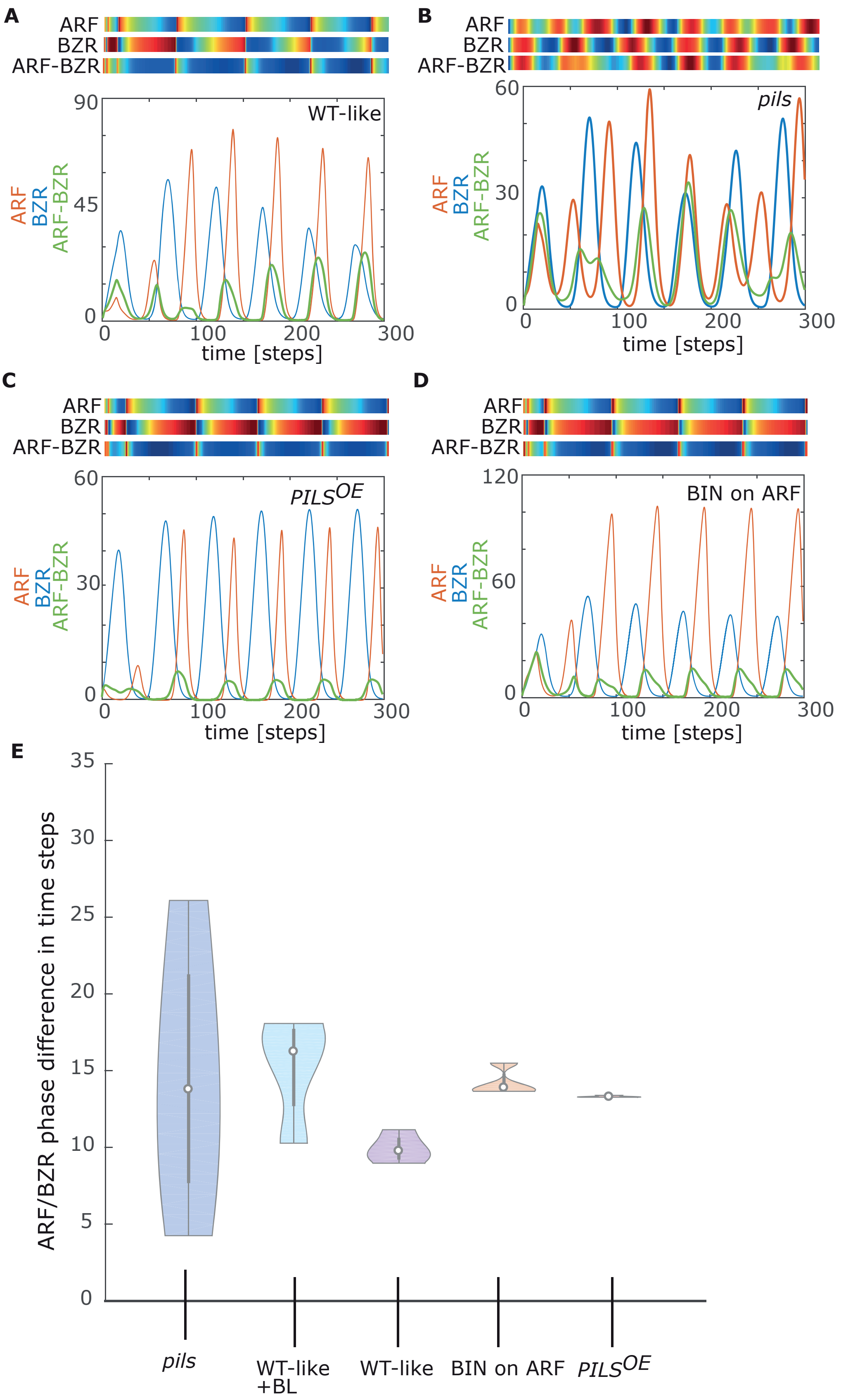
A
ARF
BZR
ARF-BZR
90
45
0
WT-like
ARF
BZR
ARF-BZR
0
100
200
300
time [steps]
B
ARF
BZR
ARF-BZR
60
30
0
pils
0
100
200
300
time [steps]
C
ARF
BZR
ARF-BZR
60
30
0
PILS^OE
0
100
200
300
time [steps]
D
ARF
BZR
ARF-BZR
120
60
0
BIN on ARF
0
100
200
300
time [steps]
E
ARF/BZR phase difference in time steps
35
30
25
20
15
10
5
0
pils
WT-like +BL
WT-like
BIN on ARF
PILS^OE

Figure S6. Simulations of computer model predicts the phase locking between BR and auxin signaling outputs through PILS-dependent auxin transport. Related to Figure 6.

(A-D) Model simulations with PILS (A), without PILS-dependent feedback (B) (corresponds to Figure 6A and 6B), as well as with mild (10-fold) *PILS* overexpression (C) and by including BIN2 effect on ARF activation (D). Graphs depict time-lapse for BZR dimers (blue), ARF dimers (red) and ARF-BZR heterodimer (green) levels and corresponding heat maps (blue to red) for peak responses. (E) Violin plots show phase difference between BZR and ARF oscillation, corresponding to model simulations (A-D).

Genotyping primers

pils2-2 FP: ATTGCTCAAGGTGAATCCAT   pils2-2 RP: AGACCAATCACGGTTAAACA

Salk_LB1-3: ATTTTGCCGATTTCGGAAC

pils5-2 FP: CCCTTGTTTGGATCATGGTA   pils5-2 RP: TCTTACTGCACCGAAAATGA

pils3-1 FP: AAAGGCATGACGACGGTTAC   pils3-1 RP: AAGAGCGTCTCCAAAATTTGG

bri1-301-FP: GGAAACCATTGGGAAGATCA   bri1-301-RP: GCTGTTTCACCCATCCAA

Cloning primers

pPILS3-ATTB4_FP: GGGGACAACTTTGTATAGAAAAGTTGCGGAAGCTAATTCTCTGAGACATAGC
pPILS3-ATTB1_RP: GGGGACTGCTTTTTTGTACAAACTTGCCTTTTTACTATCAACGCGAGAATC

qRT-PCR primers

PILS2_qPCR_FP: GTGATGCTTGTACTTGGTGGTATG
PILS2_qPCR_RP: AACTTGAACATTGGATCTGCTGAG
PILS3_qPCR_FP: AGGCGACCATGCAAGTGTTG
PILS3_qPCR_RP: GTGGTACAGCTAGATGACAGTGAG
PILS5_qPCR_FP: TCAGACGGTTACACTTGAAGACA
PILS5_qPCR_RP: GAAATGTAAGTCCCATGTTCACC
ACTIN2_qPCR_FP: ATTCAGATGCCCAGAAGTCTTGTTC
ACTIN2_qPCR_RP: GCAAGTGCTGTGATTTCTTTGCTCA

ChIP-qPCR primers

IAA19 ChIP-F: GGGAATTTGGTTTACCAGGATG
IAA19 ChIP-R: CTCAAGTATTGGGTTGAATTTTACTTAG
PILS2 ChIP-F: CCGCCGATTCATGCTTTTTAAT
PILS2 ChIP-R: AGACGACAAAAGGGAACGCT
PILS5 ChIP-F: TAATCAGAGGCTACGACGGC
PILS5 ChIP-R: CGAAAACAAACTAAATCCCAACTCA
UBC30 ChIP-F: CAAATCCAAAACCCTAGAAACCGAA
UBC30 ChIP-R: AACGACGAAGATCAAGAACTGGGAA

Table S1. Primers used in this study. Related to STAR Methods.
